# DksA is a conserved master regulator of stress response in *Acinetobacter baumannii*

**DOI:** 10.1093/nar/gkad341

**Published:** 2023-05-09

**Authors:** Ram P Maharjan, Geraldine J Sullivan, Felise G Adams, Bhumika S Shah, Jane Hawkey, Natasha Delgado, Lucie Semenec, Hue Dinh, Liping Li, Francesca L Short, Julian Parkhill, Ian T Paulsen, Lars Barquist, Bart A Eijkelkamp, Amy K Cain

**Affiliations:** ARC Centre of Excellence in Synthetic Biology, School of Natural Sciences, Macquarie University, Sydney, NSW2109, Australia; ARC Centre of Excellence in Synthetic Biology, School of Natural Sciences, Macquarie University, Sydney, NSW2109, Australia; College of Science and Engineering, Flinders University, Bedford Park, SA 5042, Australia; ARC Centre of Excellence in Synthetic Biology, School of Natural Sciences, Macquarie University, Sydney, NSW2109, Australia; Department of Infectious Diseases, Central Clinical School, Monash University, Victoria, Australia; ARC Centre of Excellence in Synthetic Biology, School of Natural Sciences, Macquarie University, Sydney, NSW2109, Australia; ARC Centre of Excellence in Synthetic Biology, School of Natural Sciences, Macquarie University, Sydney, NSW2109, Australia; ARC Centre of Excellence in Synthetic Biology, School of Natural Sciences, Macquarie University, Sydney, NSW2109, Australia; ARC Centre of Excellence in Synthetic Biology, School of Natural Sciences, Macquarie University, Sydney, NSW2109, Australia; Department of Microbiology, Biomedicine Discovery Institute, Monash University, Clayton, VIC3800, Australia; Department of Veterinary Medicine, University of Cambridge, Madingley Road, Cambridge CB3 0ES, UK; ARC Centre of Excellence in Synthetic Biology, School of Natural Sciences, Macquarie University, Sydney, NSW2109, Australia; Helmholtz Institute for RNA-based Infection Research (HIRI), Helmholtz Centre for Infection Research (HZI), 97080Würzburg, Germany; Faculty of Medicine, University of Würzburg, 97080Würzburg, Germany; College of Science and Engineering, Flinders University, Bedford Park, SA 5042, Australia; ARC Centre of Excellence in Synthetic Biology, School of Natural Sciences, Macquarie University, Sydney, NSW2109, Australia

## Abstract

Coordination of bacterial stress response mechanisms is critical for long-term survival in harsh environments for successful host infection. The general and specific stress responses of well-studied Gram-negative pathogens like *Escherichia coli* are controlled by alternative sigma factors, archetypically RpoS. The deadly hospital pathogen *Acinetobacter baumannii* is notoriously resistant to environmental stresses, yet it lacks RpoS, and the molecular mechanisms driving this incredible stress tolerance remain poorly defined. Here, using functional genomics, we identified the transcriptional regulator DksA as a master regulator for broad stress protection and virulence in *A. baumannii*. Transcriptomics, phenomics and *in vivo* animal studies revealed that DksA controls ribosomal protein expression, metabolism, mutation rates, desiccation, antibiotic resistance, and host colonization in a niche-specific manner. Phylogenetically, DksA was highly conserved and well-distributed across Gammaproteobacteria, with 96.6% containing DksA, spanning 88 families. This study lays the groundwork for understanding DksA as a major regulator of general stress response and virulence in this important pathogen.

## Graphical Abstract

**Graphical Abstract:**
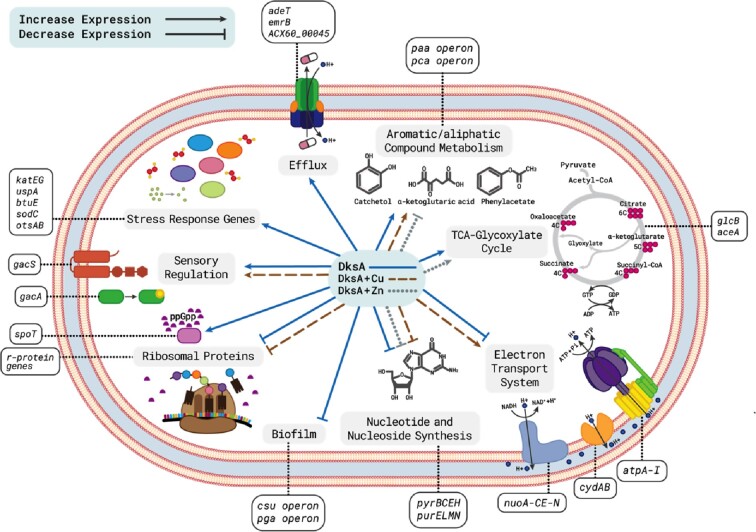
A. baumannii requires DksA as a master regulator for general stress response, metabolism and virulence. DksA has pleiotropic effects on the cell under different metal stresses.

## INTRODUCTION

*Acinetobacter baumannii*, a ubiquitous Gram-negative aerobe, has emerged as one of the most notorious human pathogens for healthcare institutions globally (1). Recently, it has been recognized by the World Health Organization as one of three top pathogens in critical need of new antibiotic therapies (2) due to its extremely high levels of antimicrobial resistance (3,4). During its lifetime, *A. baumannii* must adapt and thrive in frequently changing stresses, particularly for successful host infection and persistence in the environment (5). This pathogen displays a remarkable ability to withstand a wide range of stresses for prolonged periods, including living on desiccated surfaces for up to 250 days (6), survival in commonly used hospital disinfectants and biocides (7), as well as tolerance to stresses encountered during host infection like metal toxicity and oxidative agents (8,9). *A. baumannii*’s resilience in harsh environments can be largely attributed to its superior permeability barrier and ability to pump out toxic chemicals via efflux mechanisms (4,10–13), but how stress responses are coordinated remains poorly understood. Thus, mapping the molecular mechanisms underpinning various stress tolerance strategies in *A. baumannii* is crucial to ultimately tackle deadly clinical infections caused by this pathogen.

Bacterial stress response systems are energetically costly, and global defense mechanisms can involve a significant proportion of cell components (14). Regulation of stress response at a cellular level is largely controlled by master regulators that redistribute the limited stores of RNA polymerase to transcribe genes involved in survival and/or adaptation (14). Two major interconnected pathways coordinate bacterial stress response: the general stress response, and the stringent response. The general stress response system is regulated by an alternative sigma factor of RNA polymerase, RpoS (also called σ^38^ and σ^S^), and is well characterized in *Escherichia coli* and other common Gram-negative microbes. RpoS plays a pleiotropic role in the cell, activating genes involved in metabolism, protein processing, transport, and transcriptional regulation during starvation and other environmental challenges (14–16). Conversely, the stringent response is controlled by guanosine penta- or tetra- phosphate (p)ppGpp, a product of *relA*/*spoT* activation (17–19). However, *A. baumannii* and related species in the *Moraxella* family do not harbor a gene encoding RpoS (20,21) and thus it is likely that additional major stress regulators are active that have not yet been characterized.

To understand how stress responses are coordinated and regulated in *A. baumannii*, we investigated two biologically important metal stresses: excess copper and zinc. These metal ions are essential in all forms of life including in bacterial pathogenesis (8), yet become toxic at high concentrations (22). Host immune responses cleverly exploit both the essentiality and toxicity of copper and zinc ions to clear invading bacteria and prevent infection (8). In this study, we use a functional genomics technique, transposon insertion sequencing (23), to identify genes influencing the fitness of *A. baumannii* under copper and zinc stress, uncovering roles for efflux, membrane and envelope biogenesis. We pinpoint a previously overlooked global regulator DksA as the major coordinator of stress response in *A. baumannii*. Using transcriptomic and phenotypic profiling, we reveal how DksA acts as a switch between the two metal stressors by regulating translational machinery and metabolism. We outline a vital role of DksA in protection against other infection relevant stressors, *in vivo* host infection, maintaining mutation rates and desiccation, and retaining antibiotic resistance. Finally, phylogenetic studies confirm its wide conservation across Gammaproteobacteria and molecular studies point to key differences between DksA in *A. baumannii* and *E. coli*. Together, our results demonstrate that DksA is a crucial, conserved component of stress protection regulation in *A. baumannii*.

## MATERIALS AND METHODS

### Bacteria strains, media, and growth conditions

The wild-type *A. baumannii* strains used were ATCC 17978 (NCBI accession number: CP012004.1) and AB5075_UW (NCBI accession number: CP008706.1). The Tn*26* insertion mutant derivatives of AB5075_UW were purchased from the Manoil Laboratory (24) and used for individual growth assays to validate TraDIS results. A total of 28 mutants were used for the individual growth assays. The *dksA*::kan mutant derivative of ATCC 17978 was constructed for this study using the previously published protocol (25,26). To confirm that both the ATCC 17978 and AB5075_UW *dksA::*Tn*26* mutants contained no secondary mutations, we performed whole genome sequencing on each single gene mutant (>20× coverage on an Illumina MiSeq platform) and employed the Snippy pipeline version 4.3.6 (https://github.com/tseemann/snippy) to ensure no additional mutations were present. All primers used in this study are listed in Supplementary Table S4. All chemicals used in this study were obtained from Sigma-Aldrich (Australia) unless otherwise stated.

For routine overnight culturing of *A. baumannii* strains, a single colony from cation (calcium and magnesium ions) adjusted Mueller Hinton II (MH) agar plate (Becton Dickinson, USA), containing beef extract (3.0 g), acid hydrolysate of casein (17.5 g), starch (1.5 g) and agar (15 g) per litre of deionised water was used to inoculate 5 ml of MH broth medium and grown for 16 h at 37°C with shaking.

### Construction of transposon mutant library

The ATCC 17978 *A. baumannii* dense transposon library used in this study was constructed using the protocol as previously described (27). Briefly, transposomes were prepared by using an EZ-Tn*5* transposase (Epicentre Biotechnology) and a custom Tn*5* transposon carrying a kanamycin resistance cassette amplified from the plasmid pUT_Km (28) using the primer set as described previously (29). The transposomes (0.25 μl) were electroporated into 60 μl of freshly prepared electrocompetent cells using a Bio-Rad GenePulser II set to 1.8 kV, 25 μF and 200 Ω in a 1-mm electrode gap (Bio-Rad). For preparation of electrocompetent cells, 125 ml cultures were grown in 500 ml baffled flasks at 37°C in an Infor HT shaking incubator (Switzerland) at 200 rpm until they reached mid-log phase i.e. optical density at 600 nm (OD_600_) = 0.5. The cultures were then placed on ice for 15 min with occasional swirling before centrifugation for 10 min at 4°C, washed twice with ice-cold 10% glycerol in MilliQ (MQ) water. The washed electrocompetent cells were then resuspended with 150 μl of ice-cold 10% glycerol. The cells were resuspended in 1 ml of SOC medium and incubated at 37°C with shaking at 200 rpm for 2 h then spread on MH-agar supplemented with 7 μg/ml kanamycin (Sigma-Aldrich, Australia). Usually, 12–16 transformations were performed for each batch. Number of transformants in each batch ranged from 10000 to 50000. Approximately 250 000 mutants were collected from a total of 10 batches and stored as glycerol stocks at −80°C.

### Transposon mutant library metal stress challenge and transposon-directed insertion site sequencing (TraDIS) of mutant library

Approximately 10^9^ viable mutant cells were inoculated into 10 ml MH broth and grown at 37°C for 8 h with shaking (200 rpm). The culture (500 μl) containing approximately 10^9^ cells was sub-cultured into 10 ml fresh MH broth with or without 6 mM CuSO_4_ or 3 mM ZnSO_4_ in duplicate and grown for 16 h at 37°C with shaking (200 rpm). Genomic DNA was then extracted from approximately 10^10^ cells using the DNeasy UltraClean Microbial Kit (Qiagen) according to the manufacturer's protocol. Sequencing and analysis of the transposon mutant library were performed as described previously (30,31). The primer sets used for PCR amplification of TraDIS fragments and sequencing were described previously (29). Samples were sequenced on a HiSeq2500 Illumina sequencing platform at the Wellcome Sanger Institute, generating approximately 2 million 50 bp single-end reads per sample as previously described. TraDIS sequence reads were deposited in the European Nucleotide Archive under accession number ERP118051 and analysed using the BioTraDIS pipeline with default parameters as described previously (31). The final ATCC 17978 Tn*5* library density was >110 000 unique mutants.

### Time kill assay for the selection of copper and zinc concentrations for mutant library challenge

To identify subinhibitory concentrations of CuSO_4_ and ZnSO_4_ for treatment of the Tn*5* transposon library we performed time kill assays. Approximately 10^9^ cells from an overnight culture of ATCC 17978 was sub-cultured into 10 ml MH broth spiked with varying concentrations of CuSO_4_ (0, 3, 6, 8, 16 and 24 mM final concentration) or ZnSO_4_ (0, 3, 4, 8, 16 and 24 mM final concentration) and incubated at 37°C with shaking. At 0, 1, 2, 4, 5 and 24 h time points, 100 μl samples were taken and 10-fold serially diluted in sterile PBS and 10 μl of each dilution was then spotted on MH-agar plates. Plates were incubated at 37°C overnight and colonies were enumerated to determine the surviving cells.

### Transcriptomic analyses

Three independent cultures of *A. baumannii* strain ATCC 17978 and its Δ*dksA* mutant were grown overnight in 5 ml MH broth with shaking at 200 rpm at 37°C. The overnight cultures were diluted 200-fold in fresh MH broth and grown to mid-log phase (OD_600_ of 0.55). Each culture was divided into three flasks, with two cultures treated with either 6 mM CuSO_4_ or 3 mM ZnSO_4_ and one left untreated as a control and grown for 40 mins. RNA extraction was carried out using the miRNeasy mini kit (Qiagen) and DNA was eliminated using the TURBO DNA-free kit (Ambion Inc., USA) as per manufacturer's instructions. Libraries were constructed using a Universal Prokaryotic RNA-Seq and Prokaryotic AnyDeplete® Library preparation kit (Tecan, USA) as per to the manufacturer's instructions. The samples were sequenced on Novaseq Illumina platform, producing ∼3 million 150 bp paired-end reads per sample ∼ 25 Gbp in total. The raw sequencing data was deposited under GEO accession number GSE169081. Reads were quality controlled using FastQC and trimmed using bbduk (v38.79) with the included adapters.fa file and parameters ktrim = r k = 23 mink = 11 hdist = 1 qtrim2 = t trimq = 10 tpe tbo. Reads were then mapped using bbmap (v38.79) with parameters *k* = 13 and ambig = toss against the *A. baumannii* genome (accession CP000522) and plasmids (accessions CP000523, CP012004, CP012005), sorted using samtools (v1.6), and quantified using HTSeq (v0.12.4) with default parameters. Read counts were aggregated using a custom perl script and used as the basis for differential expression analysis. Differential expression analysis was performed in R language, using the edgeR package (v3.30.3) using the quasi-likelihood fit and test functions (glmQLFit, glmQLFTest). Genes differentially expressed, as defined by >3-fold change and *P*_adj_ <0.05, are listed in Supplementary Table S2. The function of genes in *A. baumannii* were allocated using eggNOG-mapper, and the resulting gene ontology (GO) terms and Kyoto Encyclopedia of Genes and Genomes (KEGG) pathways were used for gene set enrichment analysis (GSEA) using Fry, a fast approximation of the ROAST gene set test included in the edgeR package.

For visualising metabolic pathways in our RNA sequencing data, we used the Omics Dashboard Tool from both EcoCyc (32) and MetaCyc (33). Enrichment or depletion of metabolic pathways was then analysed using the Fisher's exact test hypothesis and significant values of <0.05. Enrichment or depletion scores (−log_10_*P* values) for each pathway in the dashboard were downloaded, and figures were created using the GraphPad Prism software (Graph-Pad Software Inc). We also downloaded tables showing list of genes from the dashboard and calculated the percentage of transcripts that increased or decreased, as shown in Figure 5D. We could not map 1338 genes out of 2470 significant up or downregulated in at least one condition due to a lack of functional annotation.

### Animal infection experiments

The *Galleria mellonella* infection experiments were performed as previously described (34). Briefly, triplicate assays of 5 larvae (200–230mg) were injected with 1 × 10^7^ cells of *A. baumannii* strains AB5075_UW or ATCC 17978 and their *dksA* mutants (total *n* = 15 per strain). Survival and health of larvae were assessed and scored every 24 h post-challenge for 7 days according to the *G. mellonella* Health Index Scoring System (35).

The *in vivo* mice model was used for enumeration of *A. baumannii* AB5075_UW and the *dksA* mutant in different host niches: blood, nasopharyngeal tissue, bronchioalveolar lavage, BAL, lung tissue, pleural cavity, PL, spleen tissue and liver. Female BALB/c mice were intranasally challenged with 2 × 10^8^ colony forming unit (CFU) and colonization was examined 24 h post-challenge using bacterial plate counting, as previously described (36). All procedures performed in this study were conducted with a view to minimize the discomfort of the animals. All experiments are approved by the University of Adelaide Animal Ethics Committee (Animal Welfare Assurance number A5491-01; project approval number S-2019-080) and were performed in strict adherence to guidelines dictated by the Australian Code of Practice for the Care and Use of Animals for Scientific Purposes.

### Mutant phenotypic assays

For all growth phenotypic assays, a single colony from Lysogeny broth (LB) agar plates was used to inoculate 5 ml of LB broth medium. Overnight cultures were diluted to an OD_600_ of 0.01 in 105 μl LB broth with or without stress treatments in 96-well plates. We supplemented ZnSO_4_ (1.5 mM), CuSO_4_ (3 mM or 5 mM) and H_2_O_2_ (0.5 mM) in LB medium for zinc, copper, and oxidative stresses respectively. For all growth assays, cultures were incubated at 37°C for 16 h with shaking at 200 rpm in a PHERAstar FS Spectrophotometer (BMG Labtech). Cell growth was monitored at 0.1 h intervals by measuring OD_600_. Growth curves were used to calculate area under curve (AUC) using the GraphPad Prism software. The difference in AUC between WT and mutants was then used as a proxy for fitness under different stress conditions.

### Mutation rate assay

Acquisition of resistance to rifampicin (Rif^R^) from rifampicin-sensitive (Rif^S^) *A. baumannii* strain AB5075_UW and its *dksA*::Tn*26* mutant (Rif^S^→Rif^R^ assay) was used to determine mutation rates. A single colony of each strain was inoculated in 5 ml MH broth and allowed to propagate overnight at 37°C with shaking at 200 rpm. The overnight cultures were diluted in 5 ml of fresh LB medium and allowed to grow to an OD_600_ of 0.6. The exponentially growing cultures were further diluted 10 000-fold and 150 μl distributed in 20 wells in 96-well plates and incubated at 37°C with shaking at 200 rpm. Aliquots (100 μl) were then plated on rifampicin MH agar plates. The concentration of rifampicin in plates was 25 μg/ml rifampicin (Sigma-Aldrich). The plates were then incubated for 24 h at 37°C to detect Rif^R^ mutant colonies. For CFU counts, aliquots of appropriately diluted cultures were plated on MH-agar plates. The mutation rates were then estimated from the number of resistant colonies per culture and the total CFU counts by using the Luria–Delbrück fluctuation test (37) and Ma-Sandri-Sarkar maximum likelihood analysis (38). The fluctuation analysis calculator (FALCOR) web tool was used for the analysis (39).

### Desiccation assay

Stationary phase (overnight grown) cells were harvested from 1 ml samples of MH cultures by centrifugation, and then were washed twice with 1 ml of sterile PBS and resuspended with MQ water. MQ water was used to prevent additional osmotic stress during drying of the cell suspensions. Cell suspensions in MQ water were adjusted to an OD_600_ of 5.0, and then 10 μl droplets of each adjusted suspension were deposited onto a plastic (polystyrene) surface in 24-well sterile plates. The samples were allowed to dry for approximately 1 h in a biosafety cabinet at ambient temperature.

To estimate the survival time of *A. baumannii* cells, dried samples were incubated in a desiccator at ambient temperature in dark. The initial number of viable cells was determined by plating 100 μl of serially diluted cultures on LB plates in triplicate. To determine viability after drying, 200 μl of PBS was added onto each dried sample. The samples were rehydrated by incubating at room temperature for 10 min, mixed thoroughly by pipetting the suspensions up and down. The suspended cultures were serially diluted in PBS and 10 μl of each dilution was then spotted onto LB agar plates. The plates were incubated at 37°C overnight. The viable cells on the dried surface were then inferred from the number of CFU recovered from each dried sample. To determine the length of survival time of desiccated samples, six dried samples were sampled every 1 or 2 days for 58 days.

### Biolog phenotypic microarray

The phenomes of *A. baumannii* ATCC 17978 and its Δ*dksA* mutant were assayed with the Biolog Phenotype MicroArrayTM (PM) system (40) to identify compounds that could serve as sole carbon sources (PM1-2; 190 compounds). Additionally, sensitivities to stress conditions (PM9-10; 192 conditions) were also tested. All phenotypic tests were performed as per manufacturer's instructions. Following inoculation, all PM plates were incubated in an OmniLog reader (Biolog) aerobically at 37°C for 48 h. Reduction of the tetrazolium-based dye (colourless) to formazan (violet) was monitored and recorded at 15 min intervals by an integrated charge-coupled device camera. The resultant data were analysed with the supplied manufacturer's software, resulting in a time-course curve for colorimetric change equating to respiration rate.

### Respiration activity assay

For respiration assays, wild-type ATCC 17978 and Δ*dksA* mutant cells in 5 ml MH broth were grown to mid-log phase (OD_600_ = 0.5) at 37°C with shaking at 200 rpm and treated with 6 mM CuSO_4_ or 3 mM ZnSO_4_ for 40 min, 1 ml cultures were centrifuged for 1.5 min and resuspended with fresh MH medium containing 0.1% tetrazolium dye and chloramphenicol (200 μg/ml). Chloramphenicol was used to prevent further protein synthesis allowing us to capture respiration status during 40 mins of copper or zinc treatment. 150 μl of cells were then transferred into 96-well culture plates. The plates were incubated in an OmniLog reader (Biolog) aerobically at 37°C for 6 h. Reduction of the tetrazolium-based dye (colourless) to formazan (violet) was monitored and recorded at 15 min intervals by an integrated charge-coupled device camera. The resultant data were analysed with the supplied manufacturer's software as in the Biolog phenotypic microarray assay.

### Serum growth inhibition assay

For the serum growth inhibition assay 10^5^ CFU in 10 μl from exponentially growing cells in MH were transferred into 100 μl of 50% serum in MH plus 0.1% tetrazolium dye in 96-well microplates. The plates were then incubated in an OmniLog reader (Biolog) aerobically at 37°C for 48 h. Reduction of the tetrazolium-based dye (colourless) to formazan (violet) was monitored and recorded at 15 min intervals by an integrated charge-coupled device camera. The resultant data were analysed with the supplied manufacturer's software.

### Biofilm formation and capsule synthesis

For biofilm formation assay we used the previously published method (41). Briefly, overnight cultures were diluted 100-fold in 100 μl LB broth in 96-well dish. Cells were then incubated for 24 h at 37°C without shaking. Bacterial cells were removed by pipetting and washed three times with PBS to remove unattached cells. We then added 125 μl of a 0.1% crystal violet (CV) aqueous solution and incubated for 15 mins at room temperature. After rinsing 3 times with water and drying for 2 h, 125 μl of 30% acetic acid in water was added to each well, incubated for 15 mins to allow complete solubilisation of CV and 125 μl of solubilised CV was transferred a new flat bottom microtiter plate. Biofilm formation was then estimated by measuring absorbance in a plate at 550 nm using 30% acetic acid solution as a blank.

For qualitative estimation of capsule levels, we used a density gradient centrifugation method as previously described (42), which is based on the effect of cell-associated capsule on bacterial density. Briefly, 1 ml of overnight grown cultures were centrifuged, washed with PBS and resuspended in 1 ml PBS. The OD_600_ of the cell suspensions was then adjusted to 1, translating to approximately 8 × 10^8^ cells/ml, and 400 μl of the cell suspensions were loaded gently on the top of a solution of 37.5% (AB5075_UW) or 47.5% (ATCC17978) Percoll in PBS. A second layer of 60% Percoll was included to aid visualisation of the cells following centrifugation. The tubes containing biphasic Percoll solution and cell suspension were centrifuged for 5 mins at 3000 x g.

### Minimal inhibitory concentration (MIC) assay

The three wild-type strains (*A. baumannii* AB5075_UW, ATCC 17978, and *E. coli* K-12) and their *dksA* single gene knockouts were streaked from frozen stocks on an MH plate overnight at 37°C. A single colony was inoculated in 10 ml of MH in a 50 ml falcon tube and shaken at 200 rpm in 37°C until an OD_600_ of 0.5 was reached. Antibiotic two-fold dilutions were prepared in triplicate in 96-well plates to a volume of 140 μl using a multichannel pipette. A 1/400 dilution was made in PBS for each of the cultures once they had reached OD_600_ of 0.5. 15 μl of the culture dilutions was dispensed into each well, bringing the final volume to 155 μl. Each plate was covered with an AeraSeal™ film (Sigma Aldrich, cat. A9224-50EA) and incubated at 37°C for overnight with shaking (200 rpm). Plates were read at OD_600,_ and MICs were reported at the lowest concentration where the majority of wells had 80% growth inhibition compared to the positive control.

### Gentamicin uptake assay

The gentamicin accumulation assay was performed using the method described previously (43). Briefly, *A. baumannii* AB5075_UW wild-type and its *dksA*::Tn*26* mutant were grown to OD = 0.6 in MH broth. Culture aliquots (500 μl) were transferred to 2 ml sterile Eppendorf tubes, and gentamicin-Texas Red conjugate was added to each sample at a final gentamicin concentration of 500 μg/ml. Reactions were protected from light and incubated for 30 min at 37°C with shaking at 200 rpm. Cells were then pelleted by centrifugation at 8000 g for 1 min, washed with 400 μl PBS, and the pellet resuspended in 1 ml DMSO and stored at −20°C prior to measurement.

Photophysical measurements were performed with a FLS980 photoluminescence spectrometer (Edinburgh Instruments) equipped with a Xe1 Xenon Arc Lamp (450 W ozone free, excitation range 230–1000 nm) for steady-state measurements. Excitation (lex) was performed at 550 nm, and emission spectra were recorded in DMSO at 28°C with 1 nm step-size, 0.1s integration time, and slit-width of Δlex = Δlem = 1.5 nm for both strains.

### Complementation of *dksA* gene homologs

Three *dksA* gene constructs were designed for complementation in *A. baumannii* cells. These include *A. baumannii* AB5075_UW full-length *dksA* (FL-Ab; amino acids 1–178), *A. baumannii* AB5075_UW truncated *dksA* (Tr-Ab; amino acids 46–178) and *E. coli* MG1655 full-length *dksA* (FL-Ec; amino acids 1–157). The gene fragments were purchased from Integrated DNA Technologies (Supplementary Table S5). Each gene fragment was cloned in the pVRL2Z (using EcoRI and NotI restriction enzymes) plasmid. Insertion of the gene fragment in the respective plasmids was confirmed via PCR followed by Sanger sequencing using sequencing primers specific for each plasmid (Supplementary Table S4).

The pVRL2 plasmid containing the cloned *dksA* sequences (FL-Ab, FL-Tr and FL-Ec) under an arabinose inducible promoter were transferred into *A. baumannii* AB5075_UW lacking *dksA* by electroporation as described previously in (44). The complementation of *dksA* (FL-Ab, FL-Tr and FL-Ec) was investigated by performing growth phenotypic assays in LB with or without the addition of added stresses. For oxidative and zinc stresses, we used 0.5 mM H_2_O_2_ and 1.5 mM ZnSO_4_ respectively, while for antibiotic stress we used rifampicin (0.4 μg/ml final concentration). For all complementation experiments we also added 0.5% arabinose in the growth medium.

### Expression, purification, and differential scanning fluorimetry for thermal melting assay of DksA

*A. baumannii* full length *dksA* was cloned into the pOPINE-3C-eGFP plasmid using NcoI and PmeI restriction enzymes. Correct insertion of the gene fragment was confirmed via PCR (primers in Supplementary Table S4) followed by Sanger sequencing. The DksA protein was expressed in *E. coli* BL21 cells and purified as previously described (45) using IMAC and SEC procedures in the presence of reducing agent tris(2-carboxyethyl)phosphine (TCEP, 1 mM). For tag cleavage (GFP-His_6_), the pooled protein fractions post-SEC were incubated with HRV-3C protease overnight at 4°C. The cleaved DksA protein was subsequently recovered using reverse IMAC. The purity of the protein was verified using sodium dodecyl sulfate polyacrylamide gel electrophoresis, showing a single band at ∼21 kDa when visualised with Coomassie blue dye.

Nanoscale differential scanning fluorimetry (nanoDSF) assays for DksA (0.5 mg/ml) were performed in duplicate in HEPES buffer (50 mM, with 300 mM NaCl, pH 8.0) supplemented with TCEP (1 mM). End-point measurement of DksA in the presence of excess zinc chloride (0.25mM), H_2_O_2_ (0.25mM) or both were made following room temperature incubation (30 min). Samples were heated over 20–95 °C at 1.5 °C min^−1^ using a Prometheus NT.48 fluorimeter (Nanotemper) controlled by PR.ThermControl. The excitation power was pre-adjusted to obtain fluorescence readings >1000 relative fluorescence units for emission at 330 nm (F330) and 350 nm (F350).

### Screening for *dksA* and *rpoS* in Gammaproteobacteria

From the 104 665 Gammaproteobacteria genomes in the Genome Taxonomy Database (GTDB) release 202 (46), 1686 genomes were selected as representative genomes – one for each genus within the class, and one for each species in the *Acinetobacter* and *Moraxella* genera. Each genome was screened for DksA (accession AKA33312.1 from *A. baumannii*) and RpoS (accession NP_417221.1 from *E. coli*) using BLASTp (47). We considered DksA present if the hit had ≥ 40% amino acid identity and ≥50% coverage; RpoS was considered present if the hit had ≥ 50% amino acid identity and ≥50% coverage. These cut-offs were selected after visualising the distribution of identity and coverage values for each gene (Supplementary Figure S4A, B). Due to high sequence similarity of DksA with TraR (Supplementary Figure S4C) and RpoS with other sigma factors, sequence similarity alone was not enough to distinguish DksA from TraR and RpoS from other sigma factors. Since DksA is relatively larger than TraR and RpoS is well known for its size of 38 kDa, we also use size information when distinguishing these proteins from other proteins. For RpoS amino acid length between 288–400 was chosen whereas for DksA ≥ 118 was chosen for cut-offs. To visualise the distribution of DksA and RpoS across the phylogeny of Gammaproteobacteria, we subset the GTDB v202 bacterial phylogeny using *ape* v5.6 (48) to select only genomes we screened. The resulting phylogeny was visualised in R using *ggtree* v3.0.4 (49) and *ggtreeExtra* v1.2.3 (50).

## RESULTS AND DISCUSSION

### Genes required for copper and zinc stress tolerance in *A. baumannii*

To identify the genetic networks important in *A. baumannii* for survival of two infection-relevant stresses, copper and zinc, we employed the fitness-based functional genomics technique, transposon directed insertion-site sequencing (TraDIS) (23,30). A high-density, random transposon library was generated in *A. baumannii* wild-type (WT) strain ATCC 17978 containing >110 000 unique Tn*5* mutants and challenged with subinhibitory levels of copper (6 mM) or zinc (3 mM) for 16 h (Supplementary Figure S1A, B). TraDIS sequencing was performed as previously described (27) and analyzed using the TraDIS Toolkit (31) to determine relative insertion frequencies. Non-essential genes whose mutants had decreased in abundance relative to untreated controls were considered necessary for metal-stress tolerance and those whose mutants increased in abundance as metal stress sensitive (using standard cut-offs of log_2_FC and *P*_adj_ < 0.05).

**Figure 1.**
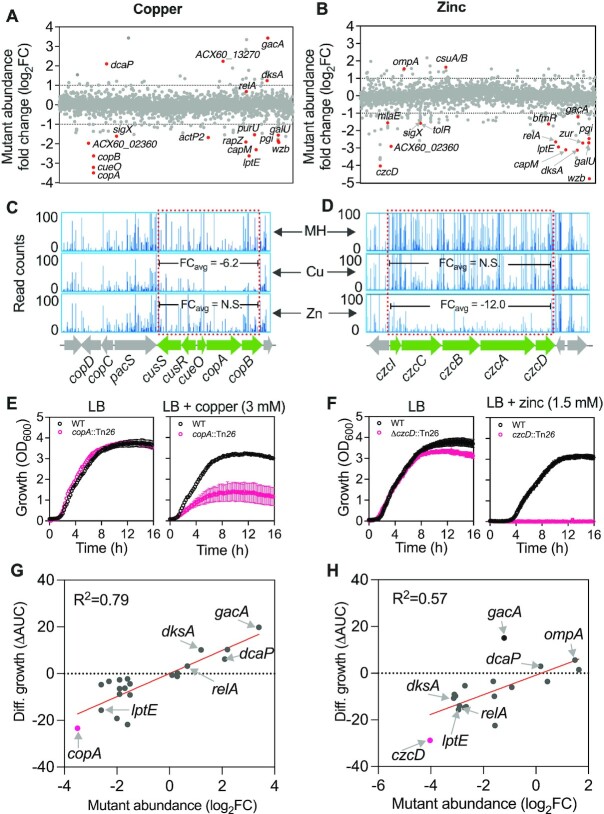
Identification and validation of *A. baumannii* genes that alter fitness under copper and zinc stresses using TraDIS. (A, B) The effect of 6 mM CuSO_4_ (**A**) and 3 mM ZnSO_4_ (**B**) on the abundance of transposon insertion mutations (differential abundance of Tn*5*, log_2_ fold change (FC)) mapping to the *A. baumannii* ATCC 17978 chromosome and plasmid pAB3 as determined by TraDIS analysis. Examples of TraDIS plots mapping at the known copper (**C**) and zinc (**D**) detoxification loci *copAB*, and *czcCBDA* of *A. baumannii*, respectively. Top panels in (C) and (D) represent insertion read counts reflecting growth of the ATCC 17978 TraDIS library in the control Mueller Hinton (MH) broth, whereas middle and bottom panels represent read counts under copper and zinc stresses, respectively. Numbers in middle panel in (C) and bottom panel in (D) represent average read count FC amongst green colored genes. N.S. stands for no significant FC compared to MH. Growth curves of the wild-type AB5075_UW (WT) and its *copA* and *czcD* mutants in presence and absence of 3 mM CuSO_4_ (**E**) and 1.5 mM ZnSO_4_ (**F**) in Lysogen broth (LB) respectively. Each data point (open black and peach circles and error bars) represents mean and standard deviation (SD) from at least three independent assays. Validation of TraDIS results using independent single gene inactivated mutants of *A. baumannii* strain AB5075_UW in copper (**G**) and zinc stress (**H**). Growth differences (measured as a difference in area under the curve, ΔAUC) between the wild-type AB5075_UW and Tn*26* insertion mutants in presence of ZnSO_4_ or CuSO_4_ was used as a proxy for fitness. Red colored gene dots with labels (Figure 1A, B) were used for validation. Each Tn*5* mutant fitness value in Figure 1A and B was calculated from two independent TraDIS experiments. See Supplementary Figure S2 and methods for further details.

The TraDIS analysis under copper stress identified 45 tolerance genes with decreased mutant fitness and 32 sensitivity genes with increased mutant fitness (Figure 1A, Supplementary Table S1). Under zinc stress, 92 tolerance genes and 31 sensitivity genes were identified (Figure 1B, Supplementary Table S1). As a sanity-check of our TraDIS genotype-phenotype screens, known metal tolerance genes were identified among the mutants with decreased abundance, such as the copper exporter *copAB* in copper treated samples (Figure 1C) and *czcABCD* transport genes in the zinc treated samples (Figure 1D) (51,52). The phenotypic growth of these representative control genes was validated using defined *copA* and *czcD* Tn*26* insertion mutants in *A. baumannii* strain AB5075_UW (24), with and without copper and zinc treatment in LB. As expected, altered growth was observed only in the presence of their respective metals (Figure 1E, F) and no drastic growth defect was observed compared to WT in untreated LB, confirming their role as specific metal resistance genes.

Next, we validated the copper and zinc growth phenotypes of a diverse collection of genes identified from the TraDIS analysis (*n* = 26) that were not previously associated with metal resistance, using single mutants in the AB5075_UW background (24) (see Supplementary Figure S2 for details of validated genes). A positive linear correlation with TraDIS data and screening with individual growth phenotype assays was detected in both copper and zinc conditions (Figure 1G, *R*^2^ = 0.79 in copper and Figure 1H, *R*^2^ = 0.57 in zinc; Supplementary Figure S2), indicating that our TraDIS results accurately predict the phenotypic impact of zinc or copper stress on individual mutants, even across two different *A. baumannii* strains.

**Figure 2.**
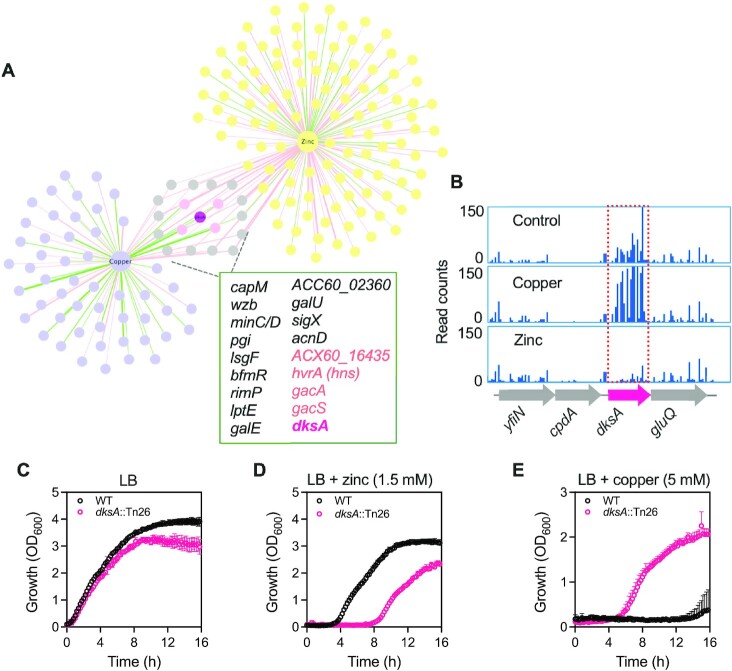
DksA has an opposite role in zinc and copper stress protection. (**A**) Network diagram showing the overlap of genes involved in tolerance and sensitivity to copper (violet) or zinc (yellow) stress. Genes represented by grey color are involved in tolerance to both copper and zinc. Pink colored genes have opposite effects under zinc and copper stresses with *dksA* in darker pink. The network analysis is based on 75 and 121 genes involved in copper and zinc stress with a significant change in mutant abundance of >1.0 log_2_ fold change and *P*_adj_< 0.05. An inset shows the list of 19 genes detected in both copper and zinc conditions. Gene with pink font have opposite effects. Cytoscape version 3.8.1. was used for network visualization (98). (**B**) The TraDIS results showing the read counts in *dksA*; control (top), 6 mM CuSO_4_ (middle) and 3 mM ZnSO_4_ (bottom). (C–E) The growth phenotype of wild-type AB5075_UW (WT) and its *dksA*::Tn*26* mutant without added stress in LB (**C**) and in presence of 1.5 mM ZnSO_4_ (**D**) and 5 mM CuSO_4_ (**E**). Data are from at least three experiments, presented as mean (open black and peach circles ± SD).

Besides the known copper and zinc efflux genes, the TraDIS analysis also identified several genes involved in other cellular functional groups (53) including cell wall/envelope/membrane biogenesis, and global regulators involved in translation and ribosome synthesis for both copper and zinc stresses (Supplementary Figure S3). These data indicated that no single pathway can fully account for the *A. baumannii* metal tolerance profile, and that multiple layers of gene regulation are required for adaptation to metal stresses. While the copper and zinc stress responses involved distinct gene networks, we also detected a subset of overlapping metal tolerance genes. For example, Tn*5* insertions in genes associated with membrane integrity and capsule synthesis (*wzb*, *galU, pgi* and *lptE*) were depleted in both copper and zinc stress (Figure 2A, Supplementary Table S1). Similarities of metal sensitivity genes were also observed, for instance, disruption of *dcaP*, an outer membrane pore forming protein for nutrient uptake (54), increased tolerance to both copper and zinc (Figure 1G, H; Supplementary Figure S2). Interestingly, disruption of *ompA* resulted in increased tolerance only to zinc stress (Figure 1H; Supplementary Figure S2).

### DksA is a pleotropic global regulator for coordinating metal stress response

The ability of the cell to defend itself from metal stresses requires not only the activation of stress-specific genes, but also global regulators that coordinate stress response. In addition to stress-specific mediators, such as metal-specific efflux pumps, we identified three global regulators that potentially play an important role in adaptation to metal stresses in *A. baumannii*. They are DksA, HvrA (homologue to H-NS) and the GacS/A two component system (Figure 2A). Interestingly these regulators showed opposite roles in copper and zinc stresses (providing tolerance to one and sensitivity in the other), potentially acting as switches. Since host immune cells exploit both the essentiality and toxicity of zinc and copper during infection (8,51), it is crucial to understand how these global regulators respond to different metal exposures and coordinate stress protection and virulence in *A. baumannii*.

While GacS/A and H-NS have been studied extensively in *A. baumannii* and are known to be dynamic coordinators of stress tolerance, virulence, motility, and antibiotic resistance (10,55,56), the molecular role of DksA is largely uncharacterized in *A. baumannii* and may play a major role in stress response. Intriguingly, our TraDIS data suggests that DksA may act in a pleotropic manner, having opposite effects in the two distinct metal conditions (Figure 2B; increased insertions in copper yet decreased insertions in zinc). Phenotypic fitness assays of a targeted *dksA*::Tn*26* mutant of *A. baumannii* strain AB5075_UW confirmed that DksA disruption is deleterious to the bacteria under zinc stress (Figure 2D), whereas it is beneficial under copper stress (Figure 2E). We also noticed that the *dksA* mutant had a comparable growth rate to WT but reached stationary phase much earlier than WT with a significantly lower growth yield (Figure 2C). Further, *relA*, which is responsible for the biosynthesis of ppGpp and mediates the stringent response, was detected as being important in zinc stress, but not copper (Supplementary Table S1). Both the TraDIS and fitness assays of a targeted *relA*::Tn*26* mutant showed that deletion of *relA* is deleterious under zinc stress (Supplementary Figure S2C), consistent with the effects of disruption of *dksA* and suggesting that ppGpp and DksA are important during metal stress. Taken together, we reasoned that DksA could play a key role in survival under multiple stresses that had not yet been fully defined in *A. baumannii*. Therefore we investigated the molecular mechanism by which DksA regulates metal stress using a suite of diverse phenotypic and genomics analyses.

### The role of DksA in virulence and colonization in animal models

**Figure 3.**
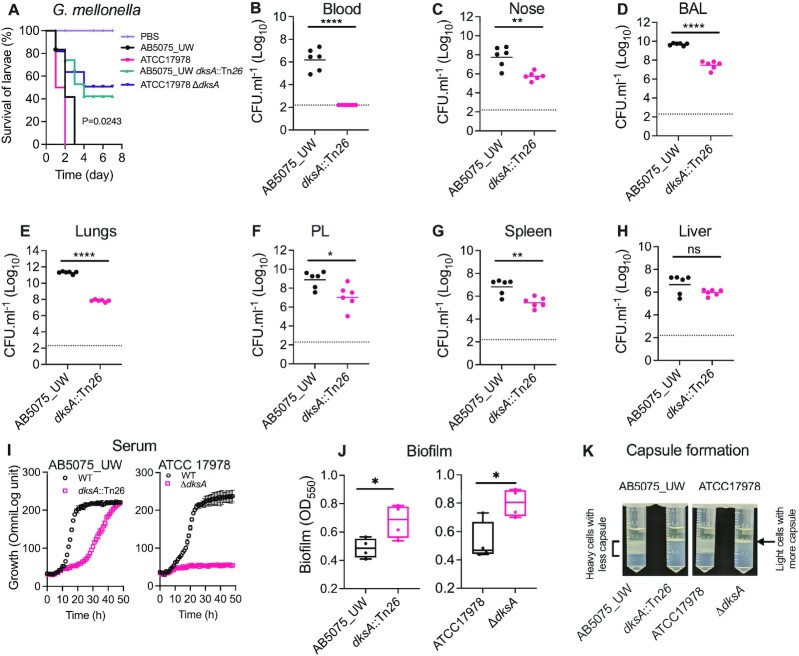
DksA-dependent virulence and niche specific colonization of *A. baumannii* and associated phenotypes. (**A**) *Galleria mellonella* larvae were injected with 1 × 10^7^ cells of *A. baumannii* strains AB5075_UW or ATCC 17978 and their *dksA* mutants. Survival of larvae was enumerated every day post-challenge for seven days. Sterile phosphate buffer saline (PBS) was used as the negative control. (B–H) Enumeration of *A. baumannii* AB5075_UW and the *dksA::*Tn*26* mutant in different host niches: blood (**B**), nasopharyngeal tissue, nose (**C**), bronchioalveolar lavage, BAL (**D**), lung tissue (**E**), pleural cavity, PL (**F**), spleen tissue (G) and liver (H). Six Female BALB/c mice were intranasally challenged with 2 × 10^8^ CFU and colonization was examined 24 h post-challenge. (**I**) Growth and respiration in presence of 50% human serum in Mueller Hinton broth. Each data point represents the mean of at least three biological triplicates (±SD). (**J**) Box and whiskers plots (min to max with all data points) showing estimates of crystal violet-based biofilm from four independent experiments. (**K**) Density gradient qualitative estimation of capsule. For significant differences between WT and mutant strains in mouse infection and biofilm formation assays, a one-way ANOVA statistical analyses were performed; * *P* < 0.05, ** *P* < 0.01, *** *P* < 0.001, **** *P* < 0.0001, and ns = not significant.

The role that DksA plays in *A. baumannii* virulence was initially tested by employing the *Galleria mellonella* wax-moth insect model, which has been shown to be an effective *in vivo* platform for molecular pathogenicity studies (57). Infection assays of *G. mellonella* using two different strains of *A. baumannii*, ATCC 17978 and AB5075_UW and their respective *dksA* mutants, were performed in triplicate batches of larvae, as previously described (34). The *dksA* mutants of both *A. baumannii* strains killed significantly fewer larvae than their WT strains, indicating that an intact DksA is required for virulence (Figure 3A). These results spurred us to investigate the role of DksA in infection of a mammalian host. For this, we intranasally challenged BALB/c mice with *A. baumannii* strain AB5075_UW or its *dksA*::Tn*26* mutant derivative. After 24 h the mice were sacrificed, organs were removed, and bacterial load counted. Strikingly, *dksA* mutant cells could not be recovered from the blood of any mice (<10^2^ cells/ml), compared to 2.5 × 10^6^ cells/ml for the WT (Figure 3B). For all tissues (Figure 3C–G) the *dksA* mutant could still colonize but showed a significant reduction in bacterial load compared to the WT, except for the liver (Figure 3H). Recovery of the *dksA* mutant from the respiratory tract (nose, bronchoalveolar and lung tissue), was at least 2 orders of magnitude lower than that seen for WT cells (Figure 3C–E). Our results also confirm the previously published INSeq transposon insertion sequencing based study in which abundance of *dksA* insertion was decreased following mouse lung infection (58). A recent study using a mouse model also showed that DksA is required for *A. baumannii* infection in the lungs but, in contrast to our animal model and serum sensitivity results, no difference in infection rates in the blood survival was observed (59).

To further understand the differences in the observed lack of ability of the *dksA* mutant to survive in the blood compared to other tissues, we performed *in vitro* virulence assays on both *A. baumannii* strains (ATCC 17978 and AB5075_UW) and their *dksA* mutants. First, we tested the mutants’ ability to propagate in human serum, which we found was greatly reduced for both *dksA* mutant strains (Figure 3I), with ATCC17978 showing an inability to grow in serum. Next, we tested the mutants’ ability to form biofilm and produce capsule and found that both were increased compared to WT (Figure 3J, K). AB5075_UW is known to produce a thick protective capsule (60) and this may be one of the reasons that its *dksA* mutant still retains partial survival in serum. Taken together, these data show that DksA is required for serum resistance and ultimately to infect the bloodstream, but it seems to repress other virulence determinants, such as biofilm. We speculate that the increase in biofilm density resulting from *dksA* disruption is what allows this mutant to retain the ability to colonize tissue, albeit not as well as WT despite being undetectable in the blood. This may be consistent with a planktonic lifestyle predominating in the blood, where enhanced biofilm formation of the *dksA* mutant may not aid colonization.

### Transcriptomics to define the DksA-dependent stress response on a molecular level

To identify the molecular mechanism underlying the dynamic role of DksA in stress protection and virulence in *A. baumannii*, we conducted RNA-sequencing (RNAseq) on the ATCC 17978 Δ*dksA* mutant and WT, treated with or without copper or zinc stress. Differential expression of 13.2% (504) of the total genes in the ATCC 17978 genome was observed for the Δ*dksA* mutant compared to WT, indicating that loss of DksA affects a large proportion of genes, even without stress induction (using a cut-off of log_2_FC > 1.5 change and *P*_adj_ < 0.05, Supplementary Table S2). The expression levels of the two adjacent genes either side of *dksA* (*nudF, cpdA and gluQ, ftsW*) showed no significant expression change in our transcriptomic analysis, indicating that there were no polar effects arising from deletion of *dksA*. Under copper and zinc stress, the number of differentially expressed genes increased to >1/5 of all genes (22.6% (859) genes and 23.6% (898) genes, respectively) for the Δ*dksA* mutant compared to the control, suggesting that DksA is a master stress regulator in *A. baumannii*.

**Figure 4.**
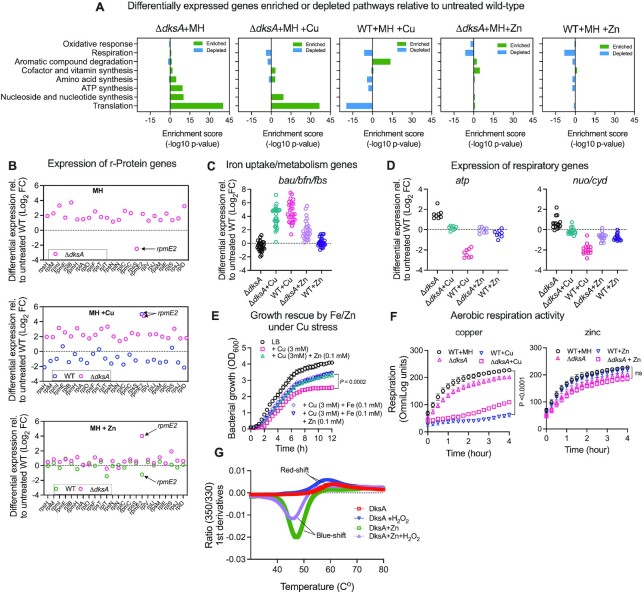
*A. baumannii* activates DksA-dependent stringent response under copper stress but not zinc stress. (**A**) Pathway enrichment analysis of genes with significant changes in expression (log_2_FC > 1.5 and *P*_adj_< 0.05) between ATCC 17978 (WT) and Δ*dksA* strains under copper and zinc stresses relative to untreated WT was performed using ‘Pathway Omics Dashboards Tool’ in the MetaCyc database, based on gene ontology (33). (**B**) Differential expression relative to untreated WT of genes involved in the synthesis of ribosomal proteins were based RNAseq data (Supplementary Table S2). (C, D) Differential expression relative to untreated WT of genes involved in the uptake and metabolism of iron (*bauABCDF*, *yciP*, *basA-J*, *bfnABC*, *fbsABDEF*) (**C**) and *atpIBEFHAGDC* operon involved in the synthesis of ATP, and NADH:quinone oxidoreductase and cytochrome bd-I ubiquinol oxidase subunits encoded by *nuoABCEFGHIJKLMN and cydAB* operons (**D**). (**E**) Growth of wild-type (WT) ATCC 17978 was measured as OD_600_ in LB in presence of indicated amount of metal ions. Error bars represent the SD of independent biological experiments (*n* = 3). (**F**) Effect of copper and zinc stress on respiration of WT ATCC 17978 and Δ*dksA* in presence of zinc and copper. Error bars represent the SD of biological independent experiments (*n* = 5). (**G**) nanoDSF curves of purified *A. baumannii* full-length DksA in the presence and absence of H_2_O_2_ (0.25 mM) or zinc chloride (0.25 mM). Error bars represent mean ± SD from two replicate analyses. *P* values in (E) and (F) are based on a two-way ANOVA. ns = not significant.

Numerous pathways were found to be heavily impacted under copper and zinc stress including translation, respiration, ATP synthesis, amino acid synthesis, aromatic compound degradation, co-factor synthesis, nucleoside and nucleotide synthesis and oxidative stress protection as in both the Δ*dksA* and WT strains (Figure 4A), based on gene ontology classification (33). Transcriptional changes in the copper-treated Δ*dksA* mutant differed markedly from the copper-treated WT and in some cases had opposite effects. For example, expression of genes involved in aromatic compound degradation increased under copper stress in the WT strain but decreased in the Δ*dksA* mutant under both treated and untreated conditions (Figure 4A, 1st, 2nd and 3rd panels from left). Conversely, expression of genes involved in protein translation decreased under copper stress in the WT strain but increased in the Δ*dksA* mutant (Figure 4A, 2nd and 3rd panels from left). The translation pathway includes ribosomal-protein (r-protein) genes; expression of these genes largely decreased in WT cells under copper stress (Figure 4B middle panel). However, in the Δ*dksA* cells expression of these genes was increased (log_2_FC 1.5–3.7) with or without copper treatment (Figure 4B, top and middle panels). Curiously, under zinc stress the transcription of r-protein genes was relatively unaffected in both WT and Δ*dksA* cells (Figure 4B, lower panel).

It is well known that nutrient limitation, such as iron, induces the stringent response in bacteria (61), which is primarily characterized by a down-regulation of r-proteins (19). In *E. coli*, DksA disrupts the interaction of RNA polymerase (RNAP) with DNA by directly binding to RNAP during the stationary growth phase, decreasing the transcription of r-proteins; thus a strain lacking DksA constitutively expresses r-proteins and r-RNAs throughout different growth phases (18,19,62). Therefore, observed downregulation of r-protein genes (induction of stringent response) in the WT cells under copper stress could be due to dysregulation of iron and zinc homeostasis, whereas it remained constitutively high in cells lacking DksA.

In fact, genes responsible for biosynthesis, uptake and export of siderophores for iron acquisition such as acinetobactin (*bauA-F*), baumannoferrin (*bfnA-L*) and fimbactin (*fbsA-Q*) gene clusters (63) were upregulated up to 180-fold under copper stress in both the Δ*dksA* and WT strains (Figure 4C). We also noted the upregulation of genes involved in zinc uptake and metabolism such as *rpmE2, zigA and tonB* (Figure 4B, middle panel and Supplementary Table S2). These genes encode the alternative ribosome subunit of the 50S protein L31, a zinc metallochaperone and zinc uptake protein respectively and are known to play crucial roles in cellular physiology during zinc limitation (64–67). In contrast, the expression of both siderophore metabolism clusters and zinc metabolism genes were not affected under zinc stress in WT (Figure 4C). We therefore hypothesized that copper stress increases synthesis of siderophores and zinc metabolism proteins that may be required to compensate for the metal starvation of the iron-sulfur (Fe–S) and zinc-dependent proteins. Consistent with this hypothesis, when we supplemented the growth medium with subinhibitory levels of ZnSO_4_ and/or FeCl_3_ in the presence of copper stress, we observed that fitness of WT *A. baumannii* improved significantly compared to the copper stress alone (Figure 4E). These results suggest that copper stress induces both iron and zinc limitation, consistent with previously reported interdependencies of copper and zinc homeostasis in this organism (68).

The stringent response in bacteria is also highly correlated with the cellular concentration of initiating nucleotide triphosphates, ATP and GTP (69–71). Most microorganisms use the Fe-S-dependent branched electron transport chain composed of NADH-quinone oxidoreductases and quinol oxidases to efficiently couple electron exchange for ATP production by the F_1_F_0_ ATPase during aerobic respiration (72,73). Since aerobic respiration contributes to more than 70% the total ATP production during bacterial growth (74), we compared respiration activities in *A. baumannii* under copper and zinc stresses as a proxy for ATP production. Both WT and Δ*dksA* exhibited similar levels of respiratory activities with or without zinc stress (Figure 4F, right panel). In contrast, copper stress resulted in a drastic reduction in respiration for WT cells (Figure 4F, left panel). A reduction of respiratory activity was also noted in the Δ*dksA* strain under copper stress, but the effect was not as severe as in WT cells. This finding was also consistent with the observed significant reduction in expression of *nuoA-N*, *cydAB* and *atpA-I* genes, which encode enzymes required for NADH:quinone oxidoreductase electron exchange, and cytochrome d ubiquinol oxidase and ATP synthesis respectively, in WT under copper stress but not in the Δ*dksA* or in treated cells (Figure 4D). Collectively, these data suggest that copper stress inhibits respiration in *A. baumannii* and DksA plays a role in exacerbating this effect under copper stress.

Recently, it has been proposed that oxidation of cysteine residues of the zinc finger is required for the allosteric activation of DksA-dependent stringent response to protect bacteria from H_2_O_2_ (75,76). Copper stress is known to generate hydroxyl radicals (^•^OH) through the Fenton-like reaction (77). We therefore hypothesized that the activation of DksA-dependent stringent response under copper stress was due to a redox-switch of DksA by the oxidation of cysteine residue. Under zinc stress, DksA remains as a zinc-bound reduced form and therefore fails to activate stress response.

To test our hypothesis, we employed nanoscale differential scanning fluorimetry (nanoDSF) analysis on the heterologously expressed and purified full-length *A. baumannii* AB5075_UW DksA protein (in presence of the reducing agent, TCEP) *in vitro*. The fluorometric technique monitors changes in intrinsic tryptophan (and tyrosine to some extent) fluorescence as a result of folding or unfolding of the protein as a function of the temperature (78). The *A. baumannii* DksA protein has only one tryptophan residue (W74), predicted to be in one of the α-helices forming the coiled-coil region (AlphaFold structure prediction, data not shown). As shown in Figure 4G, purified DksA displayed a red-shifted emission with a melting temperature (T*_M_*) of 57°C, with a mild destabilization observed following the addition of the strong oxidizing agent H_2_O_2._ In the presence of excess zinc, the assay showed a blue-shifted emission with significantly lower T*_M_* of 46°C, indicating possible allosteric changes in the chemical environment around the lone tryptophan (and potentially tyrosines) leading to the destabilization of DksA. The addition of the strong oxidizing agent H_2_O_2_ to the DksA-Zn sample did not lead to a major thermal stability shift as compared to zinc alone, supporting our hypothesis that excess zinc potentially locks and inhibits the allosteric activation of DksA.

### DksA-dependent regulation affects metabolic pathways

**Figure 5.**
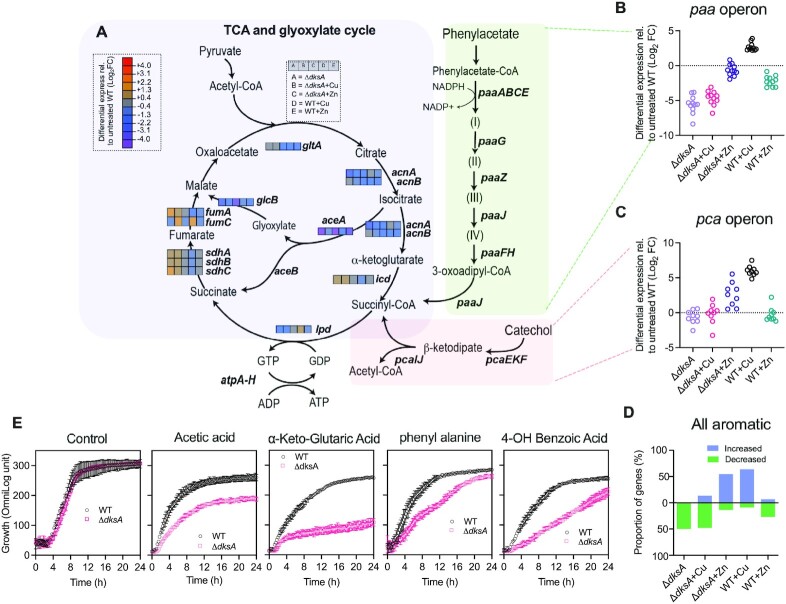
*A. baumannii* triggers DksA-dependent changes in phenylacetate, β-ketoadipate and TCA-glyoxylate pathways in response to copper stress. (**A**) Reactions and intermediates of the TCA and glyoxylate cycle (purple), phenylacetate (light green) and (pink) catechol pathways are based on BioCyc *A. baumannii* ATCC 17978 database (99). Genes (enzymes) *paaABCE* (1,2-phenylacetyl-CoA epoxidase); *paaG*, (1,2-epoxyphenylacetyl-CoA isomerase); *paaZ* (oxepin-CoA hydrolase); *paaJ* (3-oxoadipyl-CoA); *paaF* (2,3-dehydroadipyl-CoA hydratase); *paaH* (3-hydroxyadipyl-CoA dehydrogenase). Intermediate products: (I) phenylacetyl-CoA; (II) 1,2-epoxyphenylacetyl-CoA; (III) 2-oxepin-2(3H)-yli-deneacetyl-CoA; (IV) 2,3-dehydroadipyl-CoA. For simplicity, only 5 out of 9 genes are shown in the catechol pathway. (B, C) Expression of 11 genes (*paaA-H*, ACX_60–11440, *paaK*, *paaN*) in *paa* operon for phenylacetate (**B**) and 9 genes (*pcaIJFBDKCHG*) in *pca* operon for catechol catabolism (**C**) are based on transcriptomic data (Supplementary Table S2) relative to untreated *A. baumannii* ATCC 17978 WT. (**D**) Visualization of transcriptomic data in the aromatic compound degradation pathways were based on 44 genes. Bars above the line (blue) represent percentage of genes increased in expression and bars below the line (green) represent percentage of genes decreased in expression for given conditions. (**E)** Strength of aliphatic and aromatic compound utilization phenotypes of WT and its Δ*dksA* mutant were determined using Biolog Phenotype Microarray plates PM1 and PM2. The maximal kinetic curve was based on expressed OmniLog units (y-axis) over time. Metabolite utilization activity data are from two independent experiments, presented as mean ± SD.

Recently, it has been shown that activation of the stringent response determines survival success of bacteria under stress by modulating metabolic pathways (79). To test whether DksA-dependent stringent response is important for coordinating cellular metabolism in *A. baumannii*, we interrogated expression patterns of key metabolic pathways in *dksA* mutants. In *A. baumannii* metabolic pathways such as aromatic compound degradation are known to be essential for successful virulence (10). Most bacteria metabolize aromatic compounds such as catechol and protocatechuate through phenylacetate and β-ketoadipate pathways (80,81). In the phenylacetate pathway, aromatic compounds are broken down into succinyl-CoA, whereas the β-ketoadipate pathway generates succinyl-CoA and acetyl-CoA before entering the tricarboxylic acid (TCA)-glyoxylate cycle (Figure 5A). Bacterial growth on aromatic compounds, acetate, or fatty acids also require the activation of the glyoxylate shunt in the TCA and gluconeogenesis pathways (82).

In our Δ*dksA* transcriptomics, the two most differentially expressed pathways were phenylacetate and catechol pathways, encoded by genes in *paa* (*paaNABCDEFGHK*) and *pca* (*pcaIJFBDKCHG*) operons respectively (Figure 5B, C), but showed condition-specific induction. The expression of genes in the *paa* operon decreased (between 12- and 330-fold) in the Δ*dksA* cells with and without copper stress (Figure 5B). In contrast, when WT cells were treated with copper, expression of these genes increased (5- to 14-fold; Figure 5B). Whilst there was no change in expression *pca* genes with or without copper stress in the *dksA* mutant, copper stress increased expression of the *pca* operon in WT strain, mirroring effects of the *paa* operon (increased relative to untreated cells 28- to 180-fold; Figure 5C). Interestingly, under zinc stress the expression of *paa* genes were not affected in the *dksA* mutant whereas it decreased by 3- to 9-fold in the WT. In the Δ*dksA* cells, two important genes responsible for the glyoxylate shunt, *aceA* encoding isocitrate lyase and *glcB* encoding malate synthase were also downregulated (by 18- and 5-fold respectively; Figure 5A, Supplementary Table S2). Consistent with the copper stress impacting iron homeostasis, genes encoding Fe–S-dependent proteins, such as *fumC* in the TCA cycle, were upregulated under copper stress. These results indicate that a functional version of DksA is needed to activate not only specific metabolic pathways (phenylacetate and catechol) but also central metabolic pathways (TCA cycle) during metal stress in *A. baumannii*.

The mechanism(s) by which DksA induces *paa* and *pca* operons during copper stress remains unclear. However, it has been proposed that GacS/GacA two-component system operates as a switch between primary and gluconeogenic metabolites in number of bacteria (83). Additionally, carboxylic acids such as acetate and propionate have been shown to be an environmental cue for the GacS/A system (84,85). We found that expression of *gacA* was decreased in the Δ*dksA* mutant (at an average of 3.6-fold) with or without copper stress. The expression of *gacA* was not affected under both copper and zinc stresses in the WT strain (Supplementary Table S2). Thus, these data suggest that DksA controls major metabolic pathways under metal stresses by regulating the GacS/A two component signaling system.

To test whether DksA is functionally important for catabolism of substrates associated with the TCA cycle and its glyoxylate shunt, we performed Biolog phenotypic arrays that calculate bacterial respiration rates on 192 carbon sources over time (Biolog MicroArrays PM1 and PM2), as described previously (40,86). In line with expression data, the Δ*dksA* mutant showed significant growth defects in substrates (acetic and ketoglutaric acid), and aromatic carbon sources requiring the *paa* and *pca* operons such as phenylalanine and 4-hydroxy benzoic acid (Figure 5E). Taken together, the expression data of all aromatic compound-associated genes and the phenotypic growth assays suggest that DksA acts as a transcriptional switch for regulating secondary gluconeogenic pathways under stringent conditions (Figure 5D).

### Confirmation of the essential role that DksA plays in general stress response

**Figure 6.**
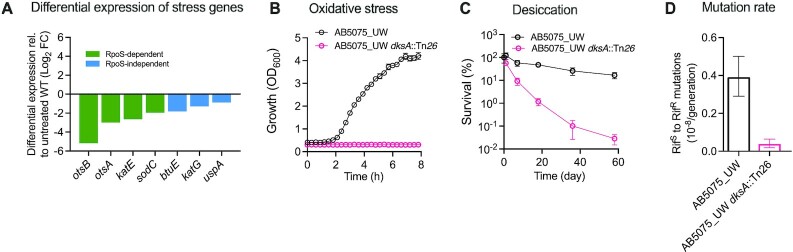
DksA regulates general stress response genes. (**A**) Differential expression of genes involved in trehalose biosynthesis and reactive oxygen species-quenching enzymes in Δ*dksA* mutant of ATCC17978 relative to WT (log_2_FC): *otsA/B* (trehalose biosynthesis); *katE* (monofunctional catalase); *sodC* (periplasmic superoxide dismutase); *btuE* (glutathione peroxidase); *katG* (bifunctional hydroperoxidase); *uspA* (universal stress protein A). Data presented are based on RNA-seq, see Supplementary Table S2 for details. (**B**) Sensitivity to oxidative stress of *A. baumannii* strain AB5075_UW and its *dksA*::Tn*26* mutant was measured as growth inhibition in LB in presence of 0.5 mM H_2_O_2_. (**C**) Survival of WT and *dksA*::Tn*26* mutant of AB5075_UW following desiccation for the indicated time points. Error bars represent ± SD of independent biological experiments (*n* = 6). (**D**) Mutation rates based on rifampicin resistance (Rif^R^) mutations from rifampicin sensitive (Rif^S^) AB5075_UW and its *dksA*::Tn*26* mutant were determined by the fluctuation test as described in the methods section. Data is presented as the mean mutation with ±95% confidence intervals (*n* = 20).

To understand the role of DksA in stress protection in *A. baumannii*, we investigated the expression pattern of stress responsive genes in the Δ*dksA* mutant of *A. baumannii*. In *E. coli*, functional *rpoS* is required for expression of oxidative stress genes such as superoxide dismutase (*sodC*) and catalases (*katE* and *katG*) as well as biosynthesis of stress protectants such as trehalose (*otsA* and *otsB*) (87). As shown in Figure 6A, expression of these RpoS-dependent genes (*katE*, *sodC*, *otsA* and *otsB*) was decreased (up to 36-fold). Expression of RpoS-independent stress genes such as *btuE, katG* and *uspA* was also decreased (2- to 4-fold; Figure 6A). The observed regulation of RpoS-dependent stress genes by DksA suggests that there may be downstream overlaps in the regulation of stress-related genes orthologs between RpoS in *E. coli* and DksA in *A. baumannii*, which lacks RpoS. However, given that RpoS and DksA both regulate hundreds of genes in the *E. coli* genome (88,89), we cannot determine the true extent of the direct functional overlap between RpoS in *E. coli* and DksA in *A. baumannii*.

To test whether down regulation of stress genes has impacted bacterial ability to cope with external stresses, we investigated the phenotypic effects of disruption of *dksA* in *A. baumannii* under oxidative and desiccation stresses. Ability to cope with these stresses are paramount for both survival on dry nosocomial environments and virulence (6). Consistent with the decreased expression of oxidative stress genes, the strain lacking a functional *dksA* was unable to grow in the presence 0.5 mM of H_2_O_2_, a well-known oxidizing agent, whereas the WT cells exhibited relatively uninhibited growth at the same concentration of H_2_O_2_ (Figure 6B). Similarly, we found that the viability of the *dksA* mutant was markedly reduced within 7 days of desiccation on dry surface at room temperature and this trend continued for next 51 days (Figure 6C). After 58 days of incubation, only 0.03% of the original population of *dksA* mutants survived. The rate of dying was much slower for WT, with a significant proportion of the original population (up to 16.7%) was still viable up to 58 days (Figure 6C), suggesting that DksA is essential for survival under oxidative and desiccation stresses.

In addition to physiological adaptation, genetic adaptation also plays an important role for both the short-term survival and long-term evolution of pathogens. RpoS is known to play a crucial role in mutagenesis in *E. coli* (90,91). To test whether DksA is also involved in mutagenesis in *A. baumannii*, we compared mutation rates in the WT *A. baumannii* strain AB5075_UW and its *dksA* mutant by measuring the frequency of rifampicin resistance mutation acquisition, which is usually conferred by base pair substitution mutations in *rpoB*, a RNA polymerase subunit B (RNAP B)-encoding gene (92). We found that the *dksA* mutant *rpoB* mutation rate was almost 10-fold lower compared to the WT, with rates of 0.04 × 10^−8^ (95% CI, 0.02–0.06 × 10^−8^) and 0.39 × 10^−8^ (95% CI, 0.29–0.5 × 10^−8^) mutations per generation, respectively, as shown in Figure 6D. These results suggest that DksA is not only essential for *A. baumannii* survival under stress conditions but also plays an important role in cell mutagenesis.

### DksA is highly conserved and widely distributed across gammaproteobacteria

**Figure 7.**
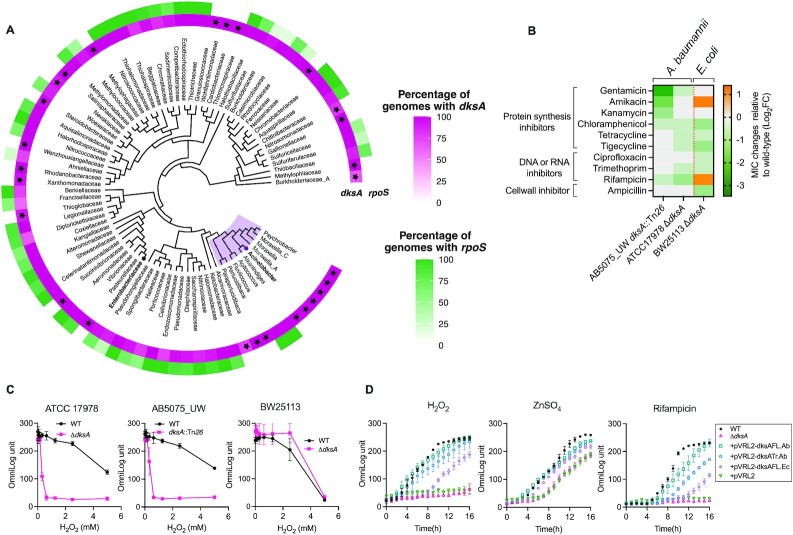
DksA is highly conserved and widely distributed across Gammaproteobacteria with distinct phenotypic effects in *A. baumannii*. (**A**) Phylogenetic tree showing distribution (%) of DksA and RpoS in Gammaproteobacteria in 88 different families and 10 genera of Moraxella, including *Acinetobacter* (in bold). See methods and Supplementary Figure S4 and Supplementary Table S6 for details. (**B**) Heatmap showing relative MIC changes of 10 different antibiotics (log_2_FC) in strains lacking *dksA* or *rpoS* in *A. baumannii* and *E. coli* against respective WTs. Negative values are more sensitive than respective WTs and positive less sensitive than respective WTs. (**C**) Sensitivity to oxidative stress in two *A. baumannii* and *E. coli* K-12 strains lacking *dksA* was estimated using series of increasing concentration of H_2_O_2_ (0–10 mM). Data is presented as the mean growth (OmniLog Unit) after 24 h from at least three independent experiments. (**D**) Phenotypic complementation of full the length (*dksA*Fl-Ab) and truncated (*dksA*Tr-Ab) of *A. baumannii* AB5075_UW and full the length *dksA* (*dksA*Fl-Ec) from *E. coli* K-12 were determined by the growth bacteria in presence of H_2_O_2_ (0.5 mM), ZnSO_4_ (1.5 mM) and rifampicin (0.4 μg/ml). See Supplementary Figure S4C and Supplementary Table S5 for details about the length (*dksA*FL-Ab) and truncated (*dksA*Tr-Ab) of *A. baumannii* AB5075_UW and full the length *dksA* (*dksA*Fl-Ec) from *E. coli* K-12.

To better understand whether the unique role of DksA in stress response is limited to *A. baumannii*, we analyzed the distribution of DksA in 1686 representative bacterial species from 88 different families across Gammaproteobacteria (Supplementary Figure S4A, B) as well as the archetypal stress-response protein RpoS. Due to a high sequence similarity of DksA with TraR (Supplementary Figure S4C) and RpoS with other sigma factors, such as RpoD (17,93), sequence similarity alone was not enough to distinguish DksA from TraR and RpoS from other sigma factors. Since DksA is relative larger than TraR and RpoS is well known for its size of 38 kDa, we also examined the length of protein sequences in addition to the sequence similarity to accurately distinguish these proteins from other similar ones using the GTDB database (see the methods section and Supplementary Figure S4C for detail). As shown in Figure 7A, DksA showed extremely high conservation and could be detected in 85 of 88 (96.6%) Gammaproteobacteria families. However, we unexpectedly observed that 34% of families did not harbor RpoS at all (starred in Figure 7A). Overall, 54.9% of 1686 representative bacterial species representatives analyzed did not harbor RpoS, although almost all Enterobacteriaceae have RpoS. These results suggest that DksA is more widely distributed amongst Gammaproteobacteria than RpoS, although its overrepresentation in Enterobacteriaceae means it is commonly studied in relation to stress response.

To test whether DksA has a direct role in providing antibiotic stress protection in *A. baumannii*, we investigated the effect of *dksA* disruption on antibiotic stress in *A. baumannii* and *E. coli* by estimating minimum inhibitory concentration (MIC) for 10 antibiotics across different classes (excluding the mutant selection marker for each strain; Figure 7B). We found that despite having very different antibiotic resistance profiles, both *A. baumannii* strains exhibited increased sensitivity to the majority of antibiotics upon disruption of *dksA* and the trend was always reduced MIC. In the *dksA* mutants, 5 out of 10 (50%) antibiotics had a decreased MIC (2 to 16-fold) for AB5075_UW and 6 out of 10 (60%) for ATCC 17978 (Figure 7B, Supplementary Table S3). The *dksA* mutant strain of *E. coli* also showed a decreased MIC for six antibiotics (60%), but unlike *A. baumannii* strains, the MIC for two antibiotics (amikacin and rifampicin) was surprisingly increased by 2- to 4-fold (Figure 7B, Supplementary Table S3), suggesting DksA has a unique role in controlling antibiotic resistance in *A. baumannii*.

The mechanisms by which DksA protects *A. baumannii* from antibiotic stress could be related to specific efflux pump activation and/or defense against endogenously antioxidants generated antibiotics such as gentamicin (94). Expression of two known resistance-related genes, ACX60_00045 and *emrB* encoding AdeT and EmrB efflux pumps, respectively, was significantly decreased (up to 5-fold) in the Δ*dksA* mutant compared to WT (Supplementary Figure S5A). AdeT is known to be involved in aminoglycoside efflux (95), and phenotypically *dksA* mutants were more sensitive to amikacin, gentamicin, and kanamycin. We hypothesized that removing DksA would affect the accumulation of antibiotics in mutant cells and investigated this using intracellular accumulation of gentamicin in the *A. baumannii dksA* mutant using a gentamicin uptake assay. We found that the *dksA* mutant cells accumulated a significantly higher level of gentamicin compared to WT cells (Supplementary Figure S5B). We did not observe a significant increase or decrease in expression of the other major efflux pumps that *A. baumannii* harbors, namely *adeIJK* and *adeABC* (Supplementary Figure S5A**)**. Taken together this suggests that DksA provides protection from stresses not only by activating stringent response but also by activating specific efflux pumps and reducing accumulation of toxic compounds inside the cells. These findings contrast to a previous study that observed significant increases in expression of efflux genes *adeB, adeIJ, abeM* and *tetA* for the *dksA* mutant of *A. baumannii* (96), which would indicate that the presence of DksA is detrimental for antibiotic resistance gene activation and conflicts with their given phenotypic resistance results. This discrepancy could be due to differences in the test conditions, for example, use of overnight culture for RNA extraction in transcription assays.

To further examine DksA’s role in stress response, we compared the ability to tolerate oxidative stress between *dksA* mutants of two *A. baumannii* strains (AB5075_UW and ATCC 17978) and *E. coli* K-12 strain BW25113 by analyzing the growth phenotypes in the presence of exogenous H_2_O_2_. The *dksA* mutants of both *A. baumannii* strains displayed a significant growth defect in the presence of as little as 0.6 mM H_2_O_2_, whereas their respective WT cells grew in up to 5 mM H_2_O_2_ (Figure 7C). However, in *E. coli*, *dksA* disruption had no impact on H_2_O_2_ survival compared to WT (Figure 7C). This data suggests that DksA is required to protect from a high level of oxidative stress is in *A. baumannii*, but is not essential for H_2_O_2_ stress protection in *E. coli*.

It is worthwhile noting that *A. baumannii* has a larger DksA (178 amino acids) compared to *E. coli* (151 amino acids) (Supplementary Figure S4C), and we suspected that the larger protein may function differently to the smaller version. To test if this difference in size contributed in observed phenotypic differences between *A. baumannii* and *E. coli*, we cloned different DksA versions into pVRL2 (44) and transformed into the *A. baumannii* AB5075_UW Δ*dksA* mutant. These versions were the full length *dksA* from *A. baumannii* (dksAFL-Ab), the truncated (dksATr-Ab) minimized version from *A. baumannii* (consensus of conserved amino acids) and the full length *dksA* from *E. coli* K-12 (dksAFL-Ec). We found that the Δ*dksA* mutant complemented with dksAFL-Ab had the greatest level of phenotypic rescue in all three test conditions (i.e. oxidative, zinc and rifampicin stresses), whereas dksAFl-Ec showed the incomplete complementation (Figure 7D). Together these results suggest that the full DksA protein is most effective in stress protection in *A. baumannii* and that the shorter *E. coli* version of DksA cannot restore function as effectively in *A. baumannii*.

## CONCLUSIONS

Our systematic genomics-based approach has uncovered DksA acts as a master regulator of stress response and virulence in *A. baumannii* and here we present the intricate details of how DksA controls stress tolerance. Our genotypic and phenotypic results allowed us to outline the pleotropic activity of DksA, acting on several core biological processes to protect the bacterial cell from stressors. The overall strategy that *A. baumannii* employs to use DksA to overcome numerous activities that are RpoS-controlled in other well-studied pathogens, like those in the *Enterobacteriaceae* family, can be rationalized in terms of its adaptive advantages. While RpoS positively regulates many genes required for stress protection, it also adversely effects the utilization of secondary carbon sources such as acetate and succinate as primary energy sources (97). In *A. baumannii*, DksA appears to play broader roles beyond regulating the direct stress genes, but also positively regulating secondary carbon metabolism and energy resources without exerting notable trade-offs associated with RpoS.

Our work provides insight into the disparate roles of DksA under seemingly similar stresses (such as copper and zinc), indicating that DksA acts as a sophisticated and sensitive molecular switch for stress response. Our study reiterates the importance of assessing gene function in less well-studied bacterial species and warns against blindly transferring function between species based solely on sequence homology, as DksA seems to play a varied role in *E. coli* and *A. baumannii* stress response. We highlighted that DksA is highly conserved in Gammaproteobacteria and is almost always present (>95%), compared to the archetypical stress response regulator RpoS, which is present far less commonly (<45%). In this study, we present an initial characterization of a conserved, DksA-mediated general stress response that provides a blueprint of a stress adaptation strategy in *A. baumannii*, which may be also appliable to other bacterial species lacking RpoS, including the key pathogens *Neisseria gonorrhoeae, Campylobacter jejuni* and *Bordetella pertussis*. Our work raises the question whether other fundamental stress response systems across diverse bacterial classes are yet to be characterized.

## DATA AVAILABILITY

The raw sequencing data was deposited under GEO accession number GSE169081. TraDIS sequence reads were deposited in the European Nucleotide Archive under accession number ERP118051.

## Supplementary Material

gkad341_Supplemental_Files
