# Supplementary material for: DksA is a conserved master regulator of stress response in Acinetobacter baumannii
Source: Nucleic Acids Res. 2023 May 9;51(12):6101–19. doi: 10.1093/nar/gkad341 (PMC10325922; doi:10.1093/nar/gkad341)
Supplement: gkad341_Supplemental_Files [file gkad341_supplemental_files.zip › Supplementary Materials_revised_FINAL_040323.pdf]

**Supplementary Materials for**

# DksA is a conserved master regulator of stress response in *Acinetobacter baumannii*

Ram P. Maharjan[1], Geraldine J. Sullivan[1], Felise G. Adams[2], Bhumika S. Shah[1], Jane Hawkey[3] Natasha Delgado[1], Lucie Semenec[1], Hue Dinh[1], Liping Li[1] Francesca L. Short[4], Julian Parkhill[5], Ian T. Paulsen[1], Lars Barquist[6,7], Bart A. Eijkelkamp[2], and Amy K. Cain[1]

**Authors' affiliations**

1. ARC Centre of Excellence in Synthetic Biology, School of Natural Sciences, Macquarie University, Sydney, NSW, 2109, Australia

2. College of Science and Engineering, Flinders University, Bedford Park, SA, Australia 5042,

3. Department of Infectious Diseases, Central Clinical School, Monash University, Victoria, Australia

4. Department of Microbiology, Biomedicine Discovery Institute, Monash University, Clayton, VIC, 3800, Australia

5. Department of Veterinary Medicine, University of Cambridge, Madingley Road, Cambridge, CB3 0ES, UK

6. Helmholtz Institute for RNA-based Infection Research (HIRI), Helmholtz Centre for Infection Research (HZI), 97080 Würzburg, Germany

7. Faculty of Medicine, University of Würzburg, 97080 Würzburg, Germany

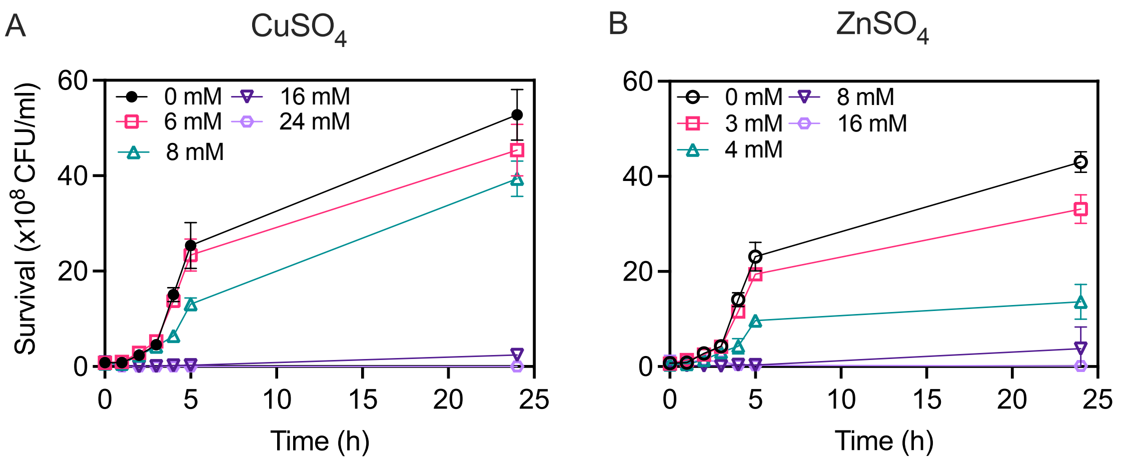


**Figure S1. Identification of the optimal copper (A) or zinc (B) concentration for TraDIS analyses**. Overnight cultures of *A. baumannii* strain ATCC 17978 were diluted to $10^8$ CFU/mL and cultured with or without indicated amount of $CuSO_4$ or $ZnSO_4$. CFUs were enumerated at various time-points following serial dilution and plating. The data are a representative of two replicate experiments.

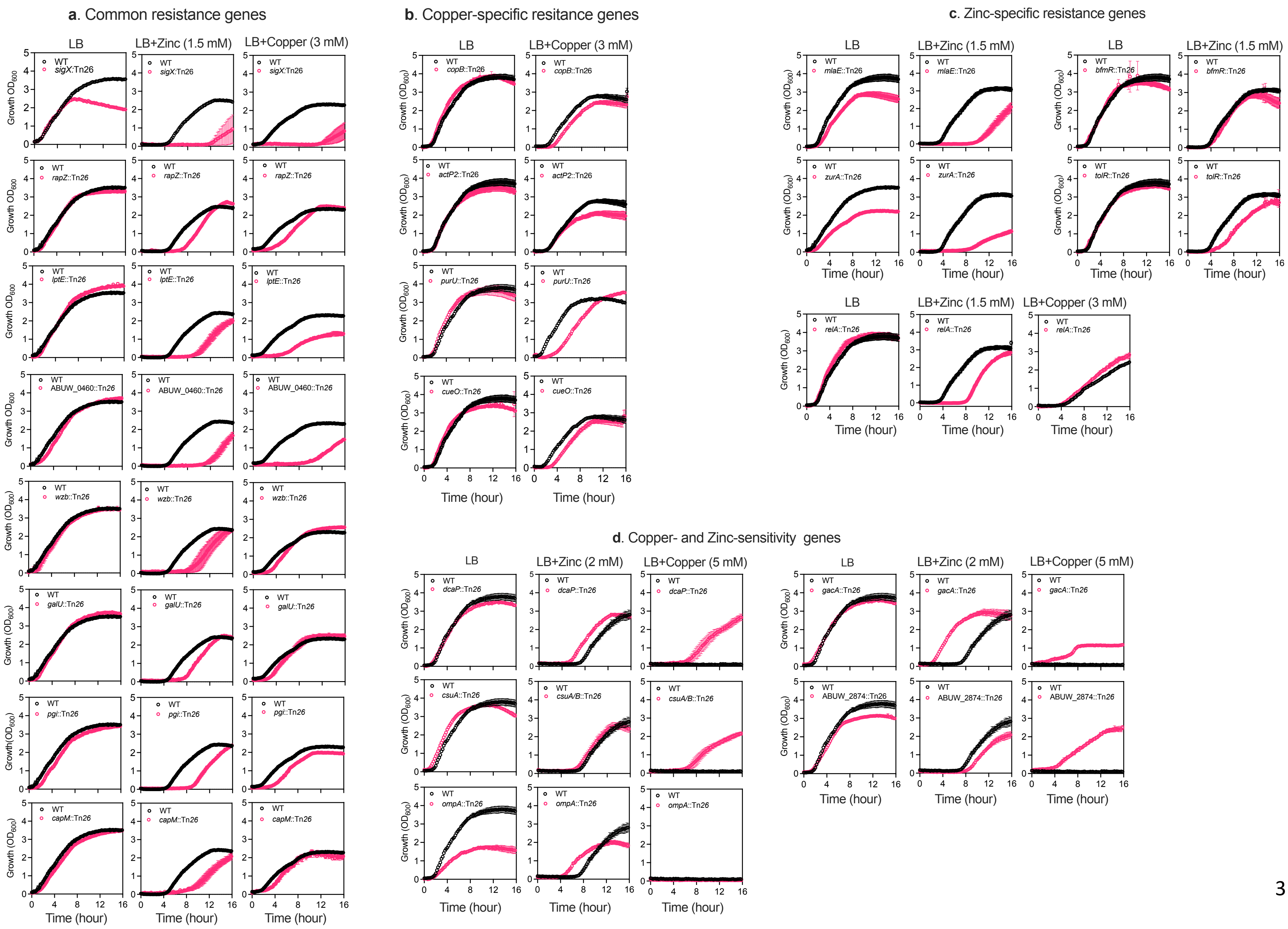
a. Common resistance genes
b. Copper-specific resitance genes
c. Zinc-specific resistance genes
d. Copper- and Zinc-sensitivity genes
LB
LB+Zinc (1.5 mM)
LB+Copper (3 mM)
LB+Zinc (2 mM)
LB+Copper (5 mM)
Growth OD600
Growth (OD600)
Time (hour)
WT
sigX::Tn26
rapZ::Tn26
lptE::Tn26
ABUW_0460::Tn26
wzb::Tn26
galU::Tn26
pgi::Tn26
capM::Tn26
copB::Tn26
actP2::Tn26
purU::Tn26
cueO::Tn26
mlaE::Tn26
zurA::Tn26
bfmR::Tn26
tolR::Tn26
relA::Tn26
dcaP::Tn26
csuA::Tn26
csuA/B::Tn26
ompA::Tn26
gacA::Tn26
ABUW_2874::Tn26

**Figure S2. Validation of genes involved in copper and/or zinc stress tolerance.** For all growth phenotypic assays, a single colony of AB5075_UW and it's Tn*26* insertion mutants from LB agar plates was used to inoculate 5 mL of LB broth medium. Overnight cultures were diluted to an optical density at 600 nm ($OD_{600}$) of 0.01 in 105 µL LB broth with or without $ZnSO_4$ (1.5 mM), $CuSO_4$ (3 mM or 5 mM). For all growth assays, cultures were incubated at 37 °C for 16 h with shaking at 200 rpm in a PHERAstar FS Spectrophotometer (BMG Labtech). Cell growth was monitored at 0.1 h intervals by measuring $OD_{600}$. Growth curves were used to calculate area under the curve (AUC) using GraphPad Prism 9.0. The difference in AUC between wild-type and mutants was and then used as a proxy for fitness under different stress conditions.

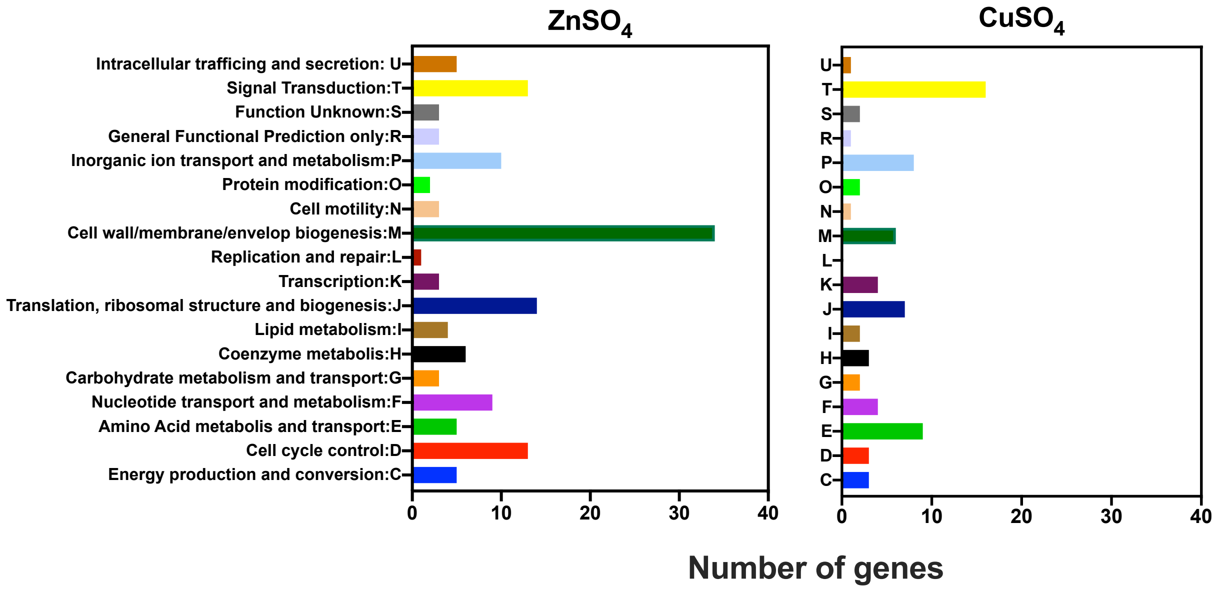


**Figure S3.** Clusters of Orthologous Groups (1) functional enrichment analysis of *A. baumannii* genes identified by TraDIS involved in zinc or copper tolerance or sensitivity. Using a cut-off of 2-fold change and $P_{adj}<0.05$, the TraDIS screen under copper stress identified 45 tolerance genes with decreased mutant fitness and 32 sensitivity genes with increased mutant fitness. Under zinc stress, 92 tolerance genes and 31 sensitivity genes were identified. For simplicity, both tolerance and sensitivity genes were pooled together.

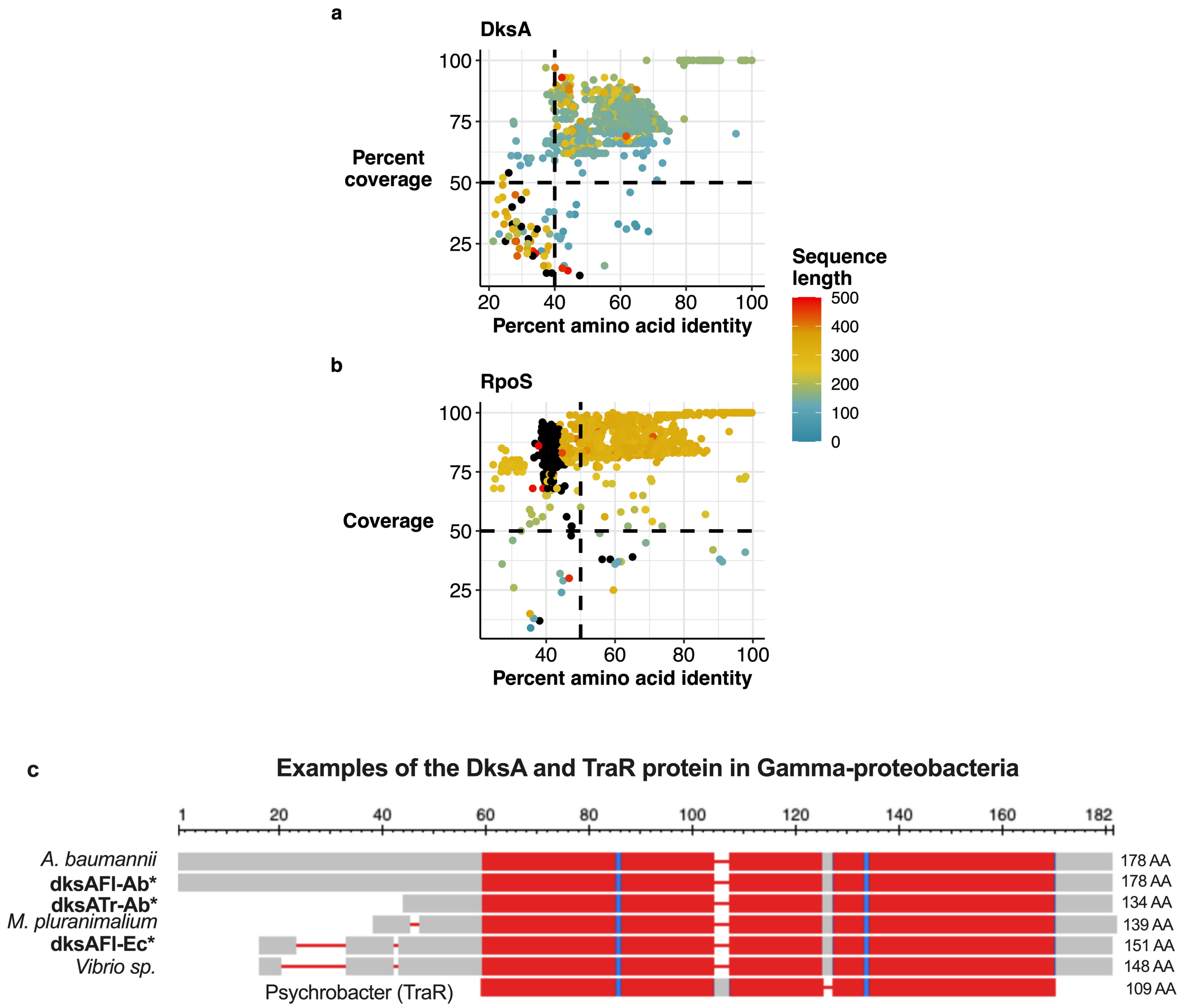


**Figure S4.** Scatter plot showing relation between sequence variability (percent identity, coverage, and size) for DksA (A) and RpoS (B) in randomly selected 1686 representative genomes across Gammaproteobacteria. Each genome was screened for protein sequences for DksA (accession AKA33312.1 from *Acinetobacter baumannii*) and RpoS (accession NP_417221.1 from *Escherichia coli*) using BLASTp (2). Dashed lines represent cut-offs used for distinguishing DksA and RpoS from other proteins. (**c**) Protein sequence alignment showing different versions of DksA and TraR in Gammaproteobacteria. Bold type entries with the asterisk sign were used for complementation of DksA in *A. baumannii*.

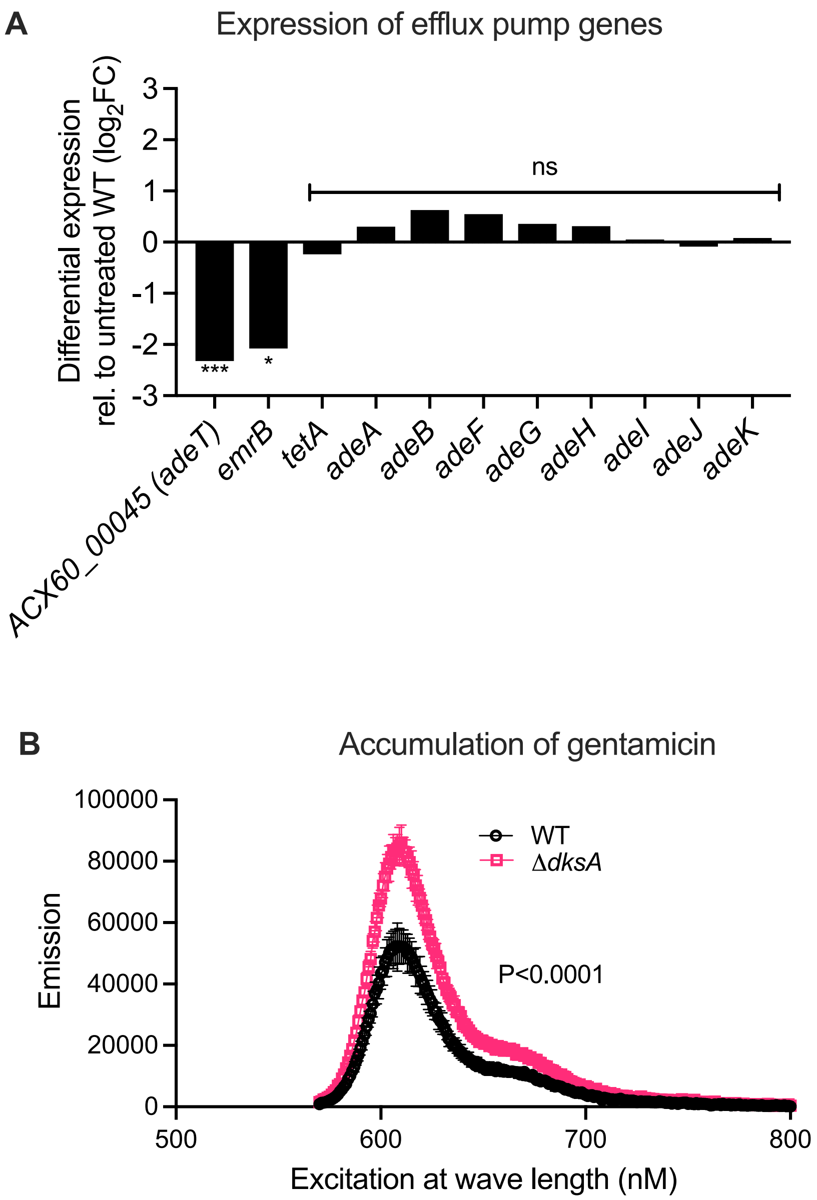


**Figure S5.** Differential expression of efflux pump genes in the Δ*dksA* mutant relative to wild-type *A. baumannii* ATCC 17978 (A). The data was based on the RNA-sequencing (RNAseq) using an Illumina platform on the ATCC 17978 Δ*dksA* mutant and wild-type. See methods for details. Differential expression relative to untreated wild type of genes involved in the synthesis of ribosomal proteins were based RNAseq data, $*P_{adj} < 0.05$, $***P_{adj} < 0.001$ and ns = not significant (see Supplementary Table 2 for detail). Accumulation of a gentamicin-Texas Red in wild-type AB5075_UW and AB5075_UW *dksA*::Tn26 cells after 30 minutes of treatment at 30 °C (B). The data were based on two biological replicates, two-tailed $P < 0.0001$.

**Table S3. Minimal inhibitory concentration (µg/mL)**

| Antibiotic | AB5075UW | | ATCC 17978 | | *E. coli* K-12 | |
|---|---|---|---|---|---|---|
| | WT | Δ*dksA* | WT | Δ*dksA* | WT | Δ*dksA* |
| Gentamicin | 2048 | 64 | 2 | 1 | 0.5 | 0.5 |
| Trimethoprim | 3000 | 3000 | 32 | 16 | 0.25 | 0.125 |
| Amikacin | 256 | 32 | 2 | 2 | 1 | 4 |
| Kanamycin | 2048 | 512 | na | na | na | na |
| Ciprofloxacin | 64 | 64 | 0.16 | 0.16 | 0.02 | 0.01 |
| Tetracycline | na | na | 1 | 0.5 | 0.5 | 0.25 |
| Ampicillin | 1024 | 1024 | 16 | 16 | 8 | 2 |
| Rifampicin | 4 | 2 | 4 | 1 | 8 | 16 |
| Chloramphenicol | 128 | 64 | 64 | 32 | 8 | 2 |
| Tigecycline | 1 | 1 | 1 | 0.5 | 1 | 0.25 |

‘na’ stands for not assayable (due to mutant selection markers for mutants).

**Table S4. Primers used in this study**

| Primer name/ purpose | Sequence (5’–3’)[a] |
|---|---|
| **[a]Construction of *dksA*::kan** | |
| *dksA*_kan_UFR_F | CATGTCATCATACAAATTAC |
| *dksA*_kan_UFR_R | GAAGCAGCTCCAGCCTACACAATCGCCATAATAGGGCATTCCT CATC |
| *dksA*_kan_DFR_F | AAGGAGGATATTCATATGGACCGGTTAATTGTGACGGTTAAG |
| *dksA*_kan_DFR_R | CAAGAAGTGAACCCTGAGAG |
| *dksA*_kan_NOL_F | GGAGTATCCACAGCTAGAA |
| *dksA*_kan_NOL_R | AGATATGTTCCGCAAAGTTG |
| Kan_FRT_F | GCGATTGTGTAGGCTGGAGCTG |
| Kan_FRT_R | G GTCCATATGAATATCCTCCTT |
| **[b]Amplification of Tn5 transposon** | |
| CV_Tn5pUTKm_F | CTGTCTCTTATACACATCTGCCACGTTGTGTCTCAAAATCTC |
| CV_Tn5pUTKm_R | CTGTCTCTTATACACATCTTCCCGTCAAGTCAGCGTAAGC |
| **[c]TraDIS PCR and Sequencing** | |
| Pf5_5’PCR | 5’AATGATACGGCGACCACCGAGATCTACACATGATGATATAT TTTTATCTTGTGCAATGTAACATC |
| Pf5_3’PCR | 3’AATGATACGGCGACCACCGAGATCTACACTCAGAATTGGTT AATTGGTTGTAACACTGGC |
| Pf5_5'Seq | C*AGAGATTTTGAGACACAACGTGGCAGATGTGT*A |
| Pf5_3'Seq | G*AGCATTACGCTGACTTGACGGGAAGATGTGT*A |
| **Construction of dksA gene fragments and confirmation** | |
| [a]pTTQ18_F | TGTGGAATTGTGAGCGGATA |
| [a]pTTQ18_R | CTGCAAGGCGATTAAGTTGG |
| [a]pVRL2Z_F | CGAGGTCGACGGTATCG |
| [a]pVRL2Z_R | TAATACGACTCACTATA |

[a] This study

[b] and [c] Primer sequences were based on previous study (3)

**Table S5.** gBlocks (Integrated DNA Technologies) gene fragment sequences used in this study

| **Description** | **Sequence**[a] |
|---|---|
| *A. baumannii* AB5075_UW full-length (FLAb) *dksA* gene fragment to clone in the pTTQ18 plasmid[b] | CAGGAAACAGCGATG**ATG**GCGAATGACAACCACAACCAAGTTTTGG<br>ATGAACATACAGAAGTTGTAGTAGAAGGTGATAAAGCTTCTGCAAA<br>ACGTGCACGTAAAGTGAAACCTAAAACTTCTGACGTAGGCTCAACT<br>GCAAGTTTATTTGGTATTGCACCTTATCAACCTAAGAAAAATGAAG<br>AGTACATGTCGGAAGGACAGCTCGAGCATTTCCGACAAATTCTGCA<br>AGCATGGAAAGCTGAATTAATGTCTGAAGTTGATCGTACTTTAAAT<br>ACGATGCAAGATGAATCAACTGCATTGCCAGACGTAAATGACCGTG<br>CTACCCAAGAAGAAGAATTTGCAATTGAATTACGTACACGTGACCG<br>TGAACGTAAATTAATTCGTAAAATCGAACAATCTCTTGAAGCGATT<br>AAAAACGAAGACTACGGTTTCTGTGAAACTTGTGGTATCGAAATCG<br>GCTTACGTCGTTTAGAAGCACGTCCAACTGCAACGTTATGTATTGA<br>CTGCAAAACTTTGGCAGAAATTAAAGAGAAGCAAAATAACGGTcgc<br>ggcagccatcaccatcatcaccat**TAA**AGCTTGGCACTGGCC |
| *A. baumannii* AB5075_UW truncated (TrAb) *dksA* gene fragment to clone in the pTTQ18 plasmid[b] | CAGGAAACAGCG**ATG**GGTATTGCACCTTATCAACCTAAGAAAAATG<br>AAGAGTACATGTCGGAAGGACAGCTCGAGCATTTCCGACAAATTCT<br>GCAAGCATGGAAAGCTGAATTAATGTCTGAAGTTGATCGTACTTTA<br>AATACGATGCAAGATGAATCAACTGCATTGCCAGACGTAAATGACC<br>GTGCTACCCAAGAAGAAGAATTTGCAATTGAATTACGTACACGTGA<br>CCGTGAACGTAAATTAATTCGTAAAATCGAACAATCTCTTGAAGCG<br>ATTAAAAACGAAGACTACGGTTTCTGTGAAACTTGTGGTATCGAAA<br>TCGGCTTACGTCGTTTAGAAGCACGTCCAACTGCAACGTTATGTAT<br>TGACTGCAAAACTTTGGCAGAAATTAAAGAGAAGCAAAATAACGGT<br>cgcggcagccatcaccatcatcaccat**TAA**AGCTTGGCACTGGCC |
| *E. coli* MG1655 full-length (FLEc) *dksA* gene fragment to clone in the pTTQ18 plasmid[b] | CAGGAAACAGCGATG**ATG**CAAGAAGGGCAAAACCGTAAAACATCGT<br>CCCTGAGTATTCTCGCCATCGCTGGGGTGGAACCATATCAGGAGAA<br>GCCGGGCGAAGAGTATATGAATGAAGCCCAGCTGGCGCACTTCCGT<br>CGTATTCTGGAAGCATGGCGTAATCAACTCAGGGATGAAGTCGATC<br>GCACCGTTACACATATGCAGGATGAAGCAGCCAACTTCCCGGACCC<br>GGTAGACCGTGCAGCCCAGGAAGAAGAGTTCAGCCTCGAACTGCGT<br>AACCGCGATCGCGAGCGTAAGCTGATCAAAAAGATCGAGAAGACGC<br>TGAAAAAAGTGGAAGACGAAGATTTCGGCTACTGCGAATCCTGCGG<br>TGTTGAAATTGGTATTCGCCGTCTGGAAGCGCGCCCGACAGCCGAT<br>CTGTGCATCGACTGCAAAACGCTGGCTGAAATTCGCGAAAAACAGA<br>TGGCTGGCcgcggcagccatcaccatcatcaccat**TAA**AGCTTGGC<br>ACTGGCC |
| *A. baumannii* AB5075_UW full-length (FLAb) *dksA* gene fragment to clone in the pVRL2Z plasmid | ATAAGCTTGATATCG**ATG**GCGAATGACAACCACAACCAAGTTTTGG<br>ATGAACATACAGAAGTTGTAGTAGAAGGTGATAAAGCTTCTGCAAA<br>ACGTGCACGTAAAGTGAAACCTAAAACTTCTGACGTAGGCTCAACT<br>GCAAGTTTATTTGGTATTGCACCTTATCAACCTAAGAAAAATGAAG<br>AGTACATGTCGGAAGGACAGCTCGAGCATTTCCGACAAATTCTGCA<br>AGCATGGAAAGCTGAATTAATGTCTGAAGTTGATCGTACTTTAAAT<br>ACGATGCAAGATGAATCAACTGCATTGCCAGACGTAAATGACCGTG<br>CTACCCAAGAAGAAGAATTTGCAATTGAATTACGTACACGTGACCG<br>TGAACGTAAATTAATTCGTAAAATCGAACAATCTCTTGAAGCGATT<br>AAAAACGAAGACTACGGTTTCTGTGAAACTTGTGGTATCGAAATCG<br>GCTTACGTCGTTTAGAAGCACGTCCAACTGCAACGTTATGTATTGA<br>CTGCAAAACTTTGGCAGAAATTAAAGAGAAGCAAAATAACGGT**TAA**<br>CGGCCGCCACCGCGG |

| | |
|---|---|
| *A. baumannii* AB5075_UW truncated (TrAb) *dksA* gene fragment to clone in the pVRL2Z plasmid | ATAAGCTTGATATCG**ATG**GGTATTGCACCTTATCAACCTAAGAAAA<br>ATGAAGAGTACATGTCGGAAGGACAGCTCGAGCATTTCCGACAAAT<br>TCTGCAAGCATGGAAAGCTGAATTAATGTCTGAAGTTGATCGTACT<br>TTAAATACGATGCAAGATGAATCAACTGCATTGCCAGACGTAAATG<br>ACCGTGCTACCCAAGAAGAAGAATTTGCAATTGAATTACGTACACG<br>TGACCGTGAACGTAAATTAATTCGTAAAATCGAACAATCTCTTGAA<br>GCGATTAAAAACGAAGACTACGGTTTCTGTGAAACTTGTGGTATCG<br>AAATCGGCTTACGTCGTTTAGAAGCACGTCCAACTGCAACGTTATG<br>TATTGACTGCAAAACTTTGGCAGAAATTAAAGAGAAGCAAAATAAC<br>GGT**TAA**CGGCCGCCACCGCGG |
| *E. coli* MG1655 full-length (FLEc) *dksA* gene fragment to clone in the pVRL2Z plasmid | ATAAGCTTGATATCG**ATG**CAAGAAGGGCAAAACCGTAAAACATCGT<br>CCCTGAGTATTCTCGCCATCGCTGGGGTGGAACCATATCAGGAGAA<br>GCCGGGCGAAGAGTATATGAATGAAGCCCAGCTGGCGCACTTCCGT<br>CGTATTCTGGAAGCATGGCGTAATCAACTCAGGGATGAAGTCGATC<br>GCACCGTTACACATATGCAGGATGAAGCAGCCAACTTCCCGGACCC<br>GGTAGACCGTGCAGCCCAGGAAGAAGAGTTCAGCCTCGAACTGCGT<br>AACCGCGATCGCGAGCGTAAGCTGATCAAAAAGATCGAGAAGACGC<br>TGAAAAAAGTGGAAGACGAAGATTTCGGCTACTGCGAATCCTGCGG<br>TGTTGAAATTGGTATTCGCCGTCTGGAAGCGCGCCCGACAGCCGAT<br>CTGTGCATCGACTGCAAAACGCTGGCTGAAATTCGCGAAAAACAGA<br>TGGCTGGC**TAA**CGGCCGCCACCGCGG |

[a] The start and the stop codon for each construct is marked in bold. A 15-bp vector overlap sequence is included at the 5’ and 3’ ends to assist with ligation independent cloning.

[b] Each construct cloned in pTTQ18 plasmid include an RGSHHHHHH epitope tag; nucleotide sequence for the tag is presented in lowercase before the stop codon for the construct.

**Legends to Supplementary Tables 1, 2, and 6**

**Table S1**. The effect of $ZnSO_4$ treatment (3 mM for 16 h) or $CuSO_4$ treatment (6 mM for 16 h) on the abundance of gene mutants (Tn*5* insertions) mapping to the *A. baumannii* ATCC 17978 chromosome and plasmid pAB3, as determined by TraDIS analysis.

**Table S2**. Differential expression of genes relative to untreated wild-type (WT) *Acinetobacter baumannii* strain ATCC 17978 in the Δ*dksA* and WT after treatment with $ZnSO_4$ (3 mM) or $CuSO_4$ (6 mM) for 40 mins, as determined by RNA sequencing.

**Table S6**. Bacterial genomes in the Genome Taxonomy Database (GTDB) used for analysis of distribution of DksA and RpoS in Gammaproteobacteria.

**References:**


1. Tatusov, R.L., Koonin, E.V. and Lipman, D.J. (1997) A genomic perspective on protein families. *Science*, **278**, 631-637.
2. Camacho, C., Coulouris, G., Avagyan, V., Ma, N., Papadopoulos, J., Bealer, K. and Madden, T.L. (2009) BLAST+: architecture and applications. *BMC Bioinformatics*, **10**, 421.
3. Fabian, B.K., Foster, C., Asher, A.J., Elbourne, L.D.H., Cain, A.K., Hassan, K.A., Tetu, S.G. and Paulsen, I.T. (2021) Elucidating Essential Genes in Plant-Associated *Pseudomonas protegens* Pf-5 Using Transposon Insertion Sequencing. *J Bacteriol*, **203**.
